# Robot-Driven Locomotor Perturbations Reveal Synergy-Mediated, Context-Dependent Feedforward and Feedback Mechanisms of Adaptation

**DOI:** 10.1038/s41598-020-61231-8

**Published:** 2020-03-25

**Authors:** Giacomo Severini, Alexander Koenig, Catherine Adans-Dester, Iahn Cajigas, Vincent C. K. Cheung, Paolo Bonato

**Affiliations:** 1 Department of Physical Medicine & Rehabilitation, Harvard Medical School, Spaulding Rehabilitation Hospital, Boston, MA USA; 2 School of Electrical and Electronic Engineering, University College Dublin, Dublin, Ireland; 3 Centre for Biomedical Engineering, University College Dublin, Dublin, Ireland; 4 Miller School of Medicine, University of Miami, Miami, Florida USA; 5 School of Biomedical Sciences, and The Gerald Choa Neuroscience Centre, The Chinese University of Hong Kong, Hong Kong, China; 6 Wyss Institute for Biologically Inspired Engineering, Harvard University, Boston, MA USA

**Keywords:** Biophysical models, Central pattern generators

## Abstract

Humans respond to mechanical perturbations that affect their gait by changing their motor control strategy. Previous work indicates that adaptation during gait is context dependent, and perturbations altering long-term stability are compensated for even at the cost of higher energy expenditure. However, it is unclear if gait adaptation is driven by unilateral or bilateral mechanisms, and what the roles of feedback and feedforward control are in the generation of compensatory responses. Here, we used a robot-based adaptation paradigm to investigate if feedback/feedforward and unilateral/bilateral contributions to locomotor adaptation are also context dependent in healthy adults. A robot was used to induce two opposite unilateral mechanical perturbations affecting the step length over multiple gait cycles. Electromyographic signals were collected and analyzed to determine how muscle synergies change in response to perturbations. The results unraveled different unilateral modulation dynamics of the muscle-synergy activations during adaptation, characterized by the combination of a slow-progressive feedforward process and a fast-reactive feedback-driven process. The relative unilateral contributions of the two processes to motor-output adjustments, however, depended on which perturbation was delivered. Overall, these observations provide evidence that, in humans, both descending and afferent drives project onto the same spinal interneuronal networks that encode locomotor muscle synergies.

## Introduction

Humans have the ability to modify their motor plan in response to changes in the walking environment, a phenomenon referred to as locomotor adaptation^1^. The study of locomotor adaption has been pursued to improve our understanding of the organization of human locomotor circuits, given the inapplicability, in humans, of the invasive techniques employed in animal models. As a case in point, studies in the lamprey, frog, turtle and cat have consistently shown that locomotor control is accomplished by rhythmic activations of lower-level spinal circuits that regulate the activities of fixed neuromuscular modules^2–6^. It has been debated if such modules exist in humans^7^, and if they encode the relative levels of activation across muscles^8^, the temporal relations among the activations of muscle groups^9^, or involve more complex structures^10,11^. A study on adaptation during forward/backward walking on a split-belt treadmill has provided evidence that human locomotion is controlled by leg-specific and forward/backward-specific independent networks^12^. These observations are compatible with a two-level organization of spinal central pattern generator (CPG) circuits where the higher level regulates the timing of activation of different muscle groups, while the lower level encodes the relative contribution of the different muscles to the task.

Despite the insights generated by the above-mentioned studies, our understanding of the neural mechanisms underlying locomotor adaptation is still limited. Specifically, how behavioral, functional and physiological processes work in concert to achieve adaptation during locomotion has remained elusive to date. In fact, there has long been uncertainty about which are the variables that drive adaptation during locomotion. Previous studies have suggested them to be related to gait stability^13^, energy expenditure^14,15^, and gait symmetry^16^. Recent studies have questioned the role of symmetry in gait adaptations, by showing asymmetric adaptive patterns that are more metabolically efficient^17^ or that better fit the kinetic requirements of the task^18^. Recent work by our group suggests a hierarchical organization of these variables with gait stability as the primary task-relevant variable^19^. Because of this hierarchy, we found that behavioral responses to perturbation are context-dependent, meaning that the presence and extent of the adaptation to a gait perturbation depends on the way in which the perturbation alters the biomechanical demands of the gait task within the environment. In previous work, we found that perturbations that alter gait stability are compensated for even at the cost of increasing energy expenditure, but perturbations that do not challenge gait stability are ignored if the necessary adjustments would lead to an increase in energy expenditure^19^. Gait symmetry is maintained if it is needed to preserve gait stability and/or minimize energy expenditure.

At the functional level, herein intended as the set of possible control strategies employed by the CNS, locomotor adaptation is generated by feedforward mechanisms^20^, although the response to the perturbation that causes the adaptive response may be marked also by feedback strategies^16,21,22^ that may depend on changes in task demands^23,24^. Feedforward, predictive, mechanisms are associated with a gradual response as the CNS generates a model of the effect of the perturbation. Feedback mechanisms are associated with reactive, faster responses^24^. How these mechanisms are triggered and combined during gait is yet a source of debate. At the neurophysiological level, adaptation has been argued to be controlled via either leg-independent^12^ or bilaterally-linked^25^ mechanisms. It is reasonable to expect that, as observed at the behavioral level, predictive/adaptive (i.e., feedforward) and reactive (i.e., feedback) responses manifested at the functional and neurophysiological levels would also be context-dependent. Understanding how these processes contribute differently to generating responses to perturbations is particularly relevant to robot-assisted gait rehabilitation, in which robot-driven forces or modifications in the walking environment are used to trigger responses aimed to achieve a physiological gait pattern. From this perspective, a dissection of how the feedforward and feedback components of automatic responses to perturbations are triggered and combined could inform the best use of devices for gait rehabilitation. As feedforward and feedback control strategies have been observed to contribute simultaneously to locomotor adaptation^21,22^, their relative input can be ideally dissected by comparing the response processes to perturbations that are expected to yield different contributions from these two mechanisms. This can be done by testing perturbations that have different biomechanical effects but that are introduced in the same general experimental paradigm.

In line with this overall objective, the aim of this study is to shed light on the mechanisms, at the functional and neurophysiological levels, of locomotor adaptation to robot-induced forces altering the gait pattern of humans. We used a robot-based adaptation paradigm^19^ and the analysis of muscle synergies^26^ to investigate the relative contributions of predictive/adaptive (i.e., feedforward) vs. reactive (i.e., feedback) locomotor control strategies. We also investigated the modular organization of the muscle synergies and assessed if their control during locomotor adaptation is unilateral or bilaterally-linked^25^. We performed two motor adaptation experiments in healthy individuals by introducing a unilateral mechanical perturbation that resulted in either an increase (X experiment) or a decrease (X_inv_ experiment) in step length. We previously observed that both perturbations trigger an adaptive response^19^. However, given the opposite biomechanical effects of the perturbations, the two related adaptation processes are expected to be obtained through different contributions of the feedforward and feedback control mechanisms. The analyses performed in this study allowed us to separate the feedforward and feedback contributions to the adaptation to both perturbations, as, respectively, slow and progressive or fast and reactive modifications in the neuromuscular controls.

The results showed, conclusively, that not only feedforward and feedback contributions can be achieved through simultaneous access to the same muscle synergies^22^, but also that they contribute to adaptation together and differently depending on the biomechanical context. Adding to the rich literature analyzing the characteristics of muscle synergies during locomotion^27^, our results provide the strongest demonstration to date that the neuromuscular adaptive control of human locomotion can be effectively described as a context-dependent sum of feedback and feedforward strategies that simultaneously shape the activation of invariant motor primitives. These results strongly suggest the presence, in humans, of a defined population of spinal interneurons regulating muscle coordination that can be accessed by both cortical and afferent drives, as observed in animal models^28^. The observations derived from this work could be used to develop new approaches to the design of robot-assisted gait rehabilitation procedures targeting specific descending- and/or afferent-driven responses in muscle synergies^29^.

## Results

**Figure 1:**
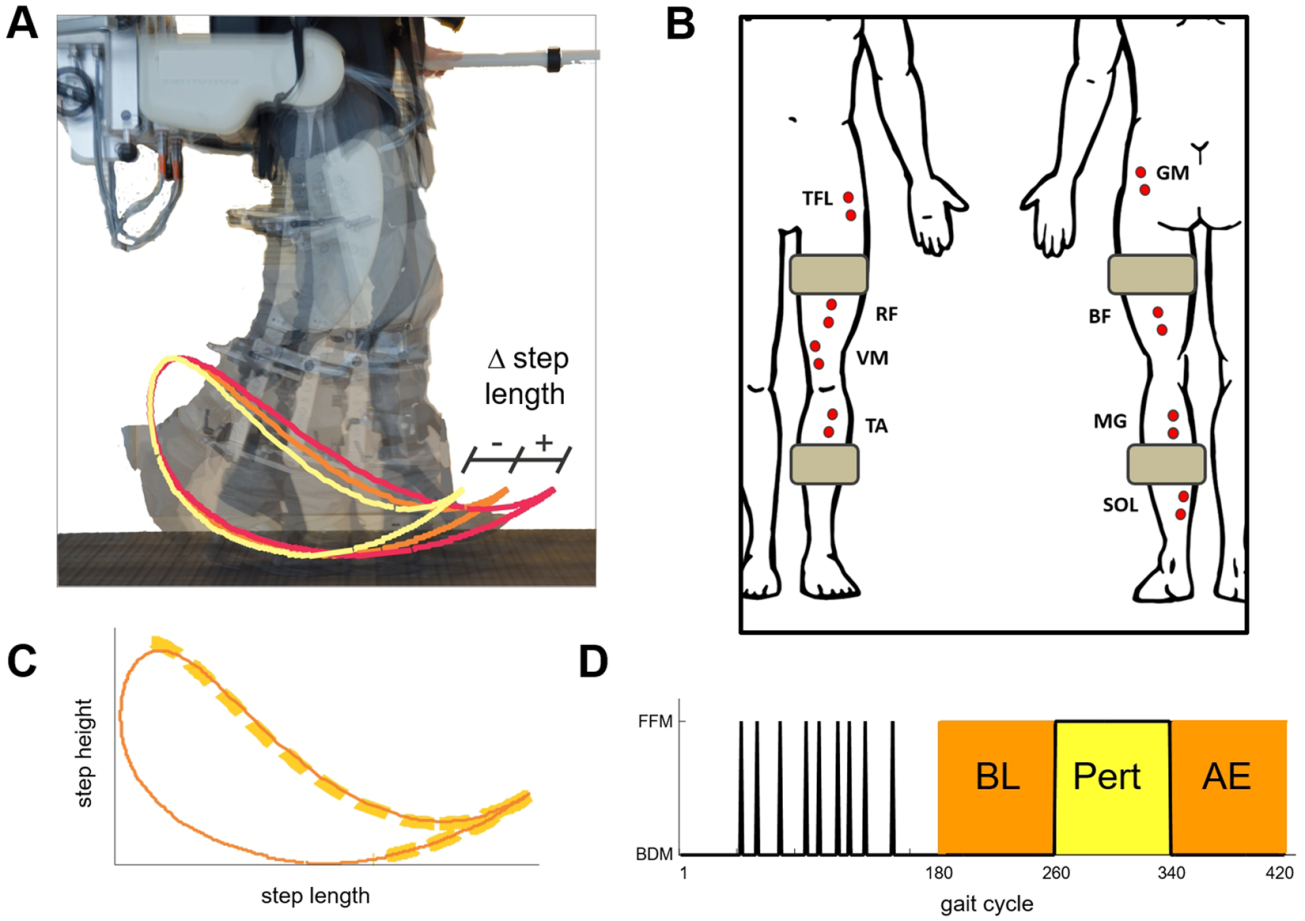
Experimental setup. (**A**) Robotic system (Lokomat, Hocoma, Switzerland) used in the experiments and graphical representation of the effects of the two perturbations on the foot trajectory. The orange, red, and light-yellow lines represent the baseline foot trajectory pattern, the effect of the X perturbation, and the effect of the X_inv_ perturbation, respectively. The robot is attached to the leg via two cuffs located roughly in the middle of the thigh and shank. **(B)** Schematic representation of the position of the EMG electrodes used during the experiments. The grey rectangles represent the positions of the cuffs linking the leg with the robotic system. **(C)** Schematic representation of the foot trajectory during the experiment (continuous line) and portion of the cycle during which the robot produced a perturbation (i.e., mid-to-terminal swing phase of the gait cycle marked by the orange bold-dashed line). **(D)** Phases of the experiment. During the habituation period (first 180 gait cycles), the system was operated in Back-Driven Mode (BDM) except for 9 Force-Field Mode (FFM) single-step perturbations randomly distributed in time. The rest of the experiment consisted of 80 BDM gait cycles (baseline - *BL*), 80 FFM gait cycles (perturbation - *Pert*) and 80 BDM gait cycles (after effect - *AE*).

Subjects walked with the lower limbs strapped to the robotic legs of a system for gait rehabilitation (Lokomat by Hocoma AG, Fig. 1A). The system was used to implement motor adaptation experiments based on a unilateral perturbation to the subjects’ right leg resulting in either an increase (X experiment) or a decrease (X_inv_ experiment) in step length^19^. Figure 1D shows a schematic representation of the experiments. Each experiment consisted of 420 gait cycles. The experiment started with a habituation phase (consisting of 180 gait cycles) followed by the Baseline (*BL*), Perturbation (*Pert*) and After-Effect (*AE*) phases of the experiment, each consisting of 80 gait cycles. During the habituation phase, the system was programmed to be transparent to the subject (Back-Driven Mode - BDM) except for 9 randomly-selected gait cycles during which the subject experienced a perturbation (Force-Field Mode - FFM). During the *BL* and *AE* phases, the robot was also programmed in BDM. During the *Pert* phase, the system was programmed in FFM and produced one of the two types of perturbation (i.e., X or X_inv_) for all the gait cycles.

### Lower-limb adaptation to two opposite perturbations

**Figure 2:**
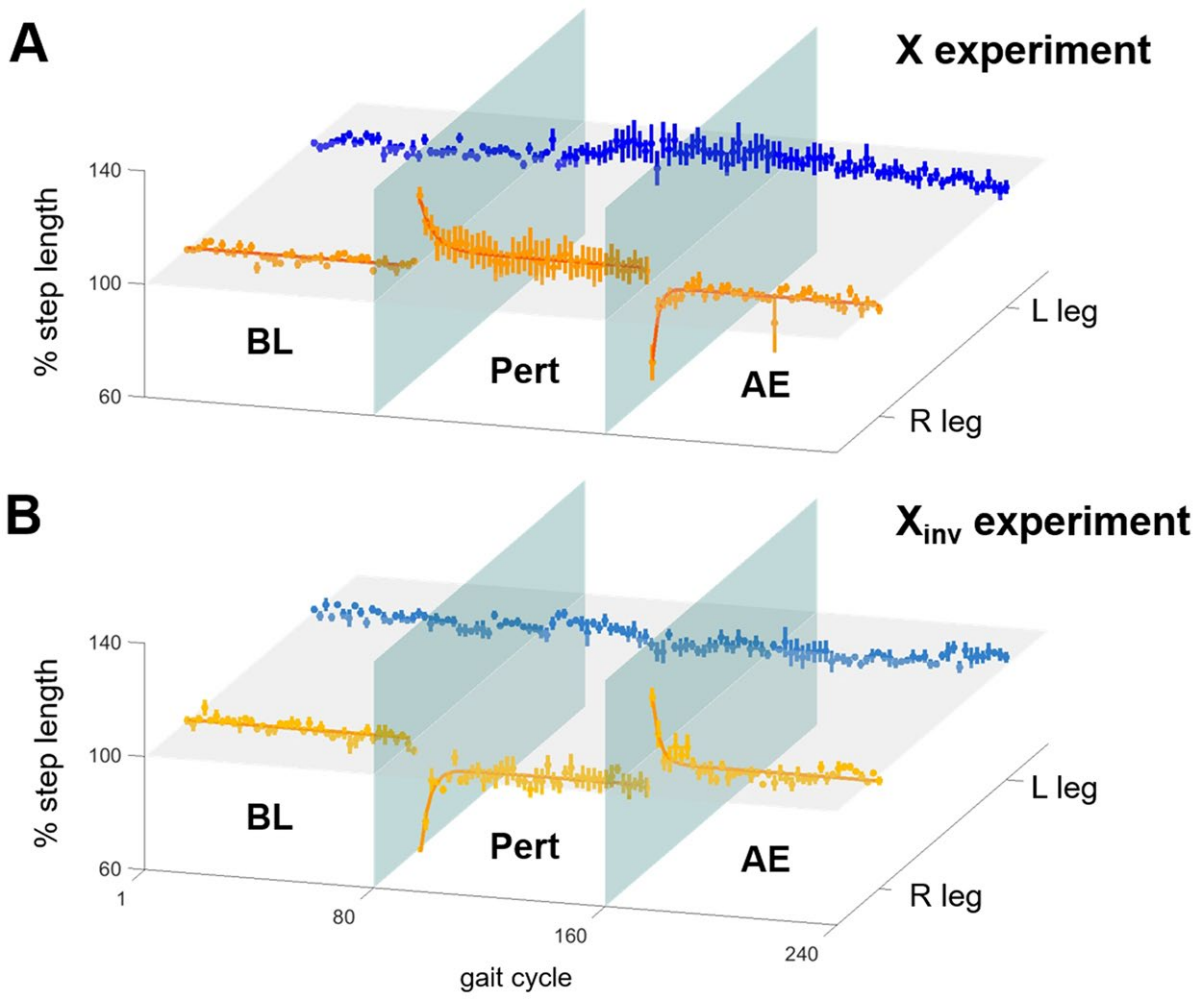
Group analysis of adaptation in step length. Percentage changes in normalized step length during the *BL*, *Pert* and *AE* phases for the left (L) unperturbed (blue/light blue) and the right (R) perturbed (orange/yellow) legs. The data is shown for **(A)** the X and **(B)** the X_inv_ experiments. Each plot represents mean and standard error of the data across subjects. The bold lines shown for the right foot plots represent the exponentials (see Eq. 3, *Materials and Methods*) fitting the group data for the *Pert* and *AE* phases of the experiments. Lines were plotted for the *BL* phase of the experiments, at values equivalent to 100% step length. The horizontal plane corresponds to the 100% step length value, while the two vertical planes represent, respectively, the beginning and end of the *Pert* phase of the experiments. For the X perturbation **(A)** we observed a ~30% increase in step length of the perturbed leg at the beginning of the *Pert* phase. The corresponding plot shows an exponential adaptation. At the beginning of the *AE* phase, we observed a decrease in step length and a rapid return to BL step length. No significant changes were observed on the contralateral side. A mirrored behavior was observed in response to the X_inv_ perturbation **(B)**.

At the onset of the X perturbation, all subjects showed an immediate step-length increase in the right, perturbed, side of 25.9 ± 6.7% (mean ± standard error) that was statistically significant (Friedman test followed by post-hoc Minimum Significance Test) (Figs. 2A and S1A). As we previously observed^19^, this change was followed by an adaptive response to the perturbation as indicated by a gradual restoration of *BL* step length in the perturbed side with a residual positive deviation (at the end of the *Pert* phase) of 5.5 ± 4.8%. This residual deviation from *BL* step length was statistically different from the deviation observed at the beginning of the *Pert* phase, but not significantly different from the *BL* step length, indicating that subjects were able to restore their baseline step length. Full adaptation to the X perturbation, estimated as 3 times the time constant of the exponential function fitting the average step-length time-course, was found to require 13.8 gait cycles (with the 95% confidence interval ranging from 10.6 to 19.9 gait cycles). At the beginning of the *AE* phase, when the perturbation was removed, a significant aftereffect, manifested as a 29.4 ± 6.7% decrease in step length in the perturbed side, was observed. All subjects then gradually returned to their *BL* step length.

Similar results were observed during the X_inv_ experiments, but opposite in the direction of step-length change (Figs. 2B and S1B). The initial response to the X_inv_ perturbation was marked by a significant decrease in step length in the perturbed side (41.2 ± 3.8%). At the end of the *Pert* phase, a residual step-length decrease of 9.7 ± 3.7% was observed in the perturbed side that was statistically different from the step length at the beginning of the *Pert* phase, but not from the *BL* step length, indicating, again, a return to the baseline step length. Full adaptation was achieved in 9.3 gait cycles (with the 95% confidence interval ranging from 7.2 to 13.3 gait cycles). A significant step-length increase in the perturbed side (28.2 ± 3.8%) was observed upon removal of the perturbation. All subjects were then able to return to their *BL* step length. No significant change was observed in the step length of the unperturbed left leg during both experiments (Figs. 2A,B and S1A,B). The time-course of adaptation for both experiments was in line with what we observed in previous experiments based on the same setup and perturbations^19^.

### Four muscle synergies reconstructed baseline electromyographic data

Muscle synergies were extracted both unilaterally (i.e., using data collected from one leg at the time) and bilaterally (i.e., using data collected from both legs). The bilateral analysis was employed to highlight the presence or absence of adaptation processes that are bilaterally-linked.

**Figure 3:**
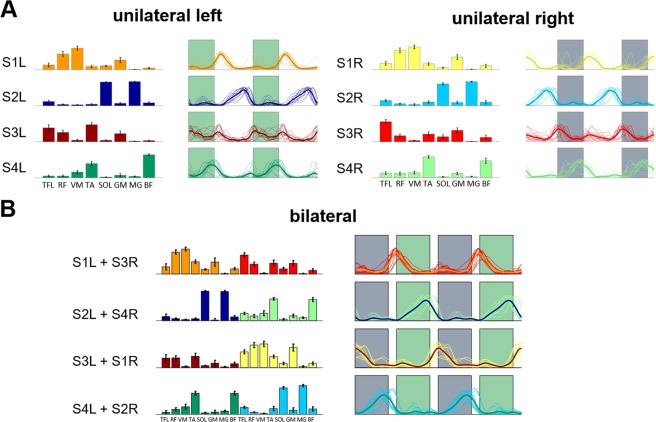
Reference synergies extracted during *BL*. Four unilateral synergies were extracted for both the left and right legs **(A)**. Weights for each synergy are represented as average and standard deviation across the two experiments of all the subjects. The activation patterns are represented both individually (thin lines) and as an average across the two experiments of all subjects (bold line). Shaded areas represent the swing phase of the left (green) and right (grey) gait cycles, respectively. Four muscle synergies were also extracted in the bilateral analysis **(B)** that appeared to result from the combination of the unilateral synergies.

Four muscle synergies were sufficient to reconstruct the amplitude modulation of the electromyographic (EMG) data during the *BL* phase with a R^2^ > 0.75 in both the unilateral and bilateral analyses (R^2^ = 0.80 ± 0.02 for the bilateral analysis; R^2^ = 0.86 ± 0.06 for the unilateral left leg analysis; R^2^ = 0.79 ± 0.04 for the unilateral right leg analysis). The muscle synergies and their corresponding activation patterns identified from the EMG recordings collected during the *BL* phase of both experiments (Fig. 3) were used as the reference synergy patterns for the X and X_inv_ experiments, and are herein referred to as *REF*_*synX*_ and *REF*_*synXinv*_, respectively. The results of the unilateral analyses (Fig. 3A) are consistent in their muscular compositions with those previously reported for a similar set of muscles^8,25^. The rectus femoris (RF) and vastus medialis (VM) muscles are the main contributors to synergy #1 (S1). This synergy is primarily active during early stance and provides stability during the loading response phase of the gait cycle. The soleus (SOL) and gastrocnemius (medial head) (MG) muscles are the main contributors to synergy #2 (S2). This synergy provides propulsion during terminal stance. The tensor fasciae latae (TFL), RF, gluteus maximus (GM), and tibialis anterior (TA) muscles are the main contributors to synergy #3 (S3). During initial swing, this synergy provides ankle dorsiflexion and hip flexion. Finally, the TA and biceps femoris (BF) muscles are the main contributors to synergy #4 (S4), which is primarily active during terminal swing and is responsible for foot landing negotiation and deceleration. In the bilateral analysis (Fig. 3B), S1 of each leg combined with S3 of the contralateral leg, while S2 combined with S4. This is due to the temporal association among the phases of the gait cycles of the two legs.

### No additional muscle synergy recruited during motor adaptation

At the level of muscle synergies, we expect adaptation to occur through one of the following mechanisms:

- Through recruitment of ≥1 additional task-specific synergies^30,31^.
- Through modification of the activation patterns of a fixed set of muscle synergies^22,32^.
- Through modification of the weights of select muscles in some or all the muscle synergies^33^.
- Through a combination of *a, b* and/or *c*^30^.

Herein, we systematically assess which of the above mechanisms may be at work during adaptation to the perturbations tested in the study (see *Materials and Methods*). We first analyzed if adaptation is associated with mechanism *a* by examining whether adaptation leads to a change in the number of synergies composing the EMG data. This was achieved by finding the dimensionality of the subspace shared between the 4 *BL* synergies (*REF*_*synX*_ and *REF*_*synXinv*_) and sets of 4 or 5 synergies for the unilateral analyses and 4, 5 or 6 synergies for the bilateral analysis that were extracted from the EMG data recorded during the last 10 gait cycles of the *Pert* phase (herein referred to as the *late-Pert* phase) when full adaptation had been attained. We reasoned that if a new synergy emerged after adaptation without altering the pre-existing four, the dimensionality of the subspace shared between the *BL* synergy set and the *late-Pert* set would increase as the number of synergies composing the latter increased. This is because the original 4 *BL* synergies can be clearly identified as shared subspace dimensions only when the new additional synergies during the *late*-*Pert* phase, if any, are properly accommodated for as extra dimensions. It is worth pointing out that we tested 4, 5 and 6 synergies for the bilateral analysis (but only 4 and 5 synergies for the unilateral analyses) to account for the possibility that two distinct unilateral synergies could emerge from the analysis of the *late-Pert* data that would not merge in a single bilateral synergy (contrary to what was observed for the *BL* phase of the experiment).

**Figure 4:**
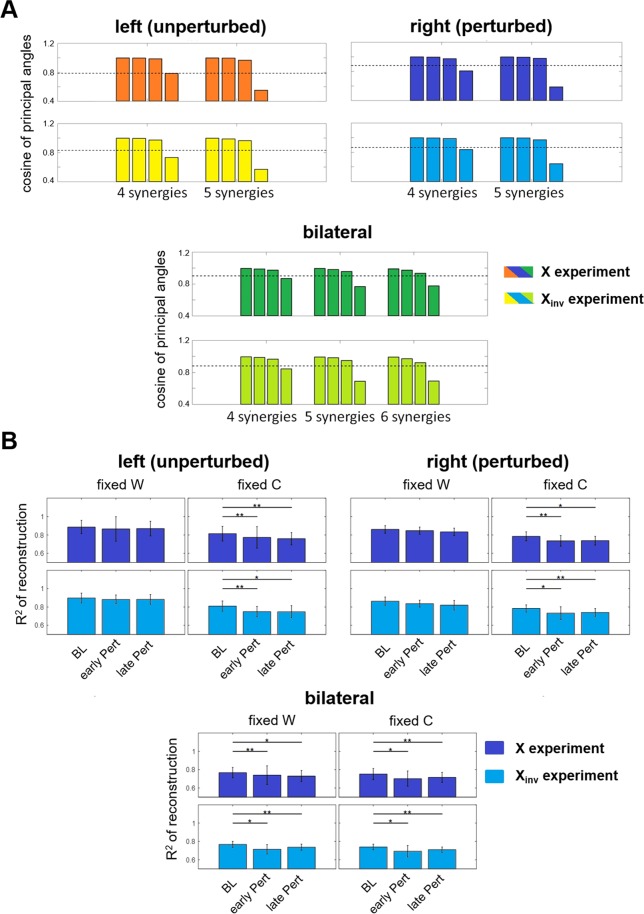
Analysis of the dimensionality of the synergy subspaces. (**A**) Bar plots of the average - across subjects - cosines of the matched principal angles between the reference synergies (extracted from the gait cycles of the *BL* phase) and the synergies extracted unilaterally for both legs during the *late*-*Pert* phase (extracted from the last 5 segments, i.e., 10 gait cycles). Results obtained by comparing 4 and 5 unilateral (top plots) and 4, 5 and 6 bilateral synergies (bottom plots). The dotted line represents the threshold value obtained through surrogate data analysis (see *Materials and Methods*). For all the analyses, we observed a consistent shared subspace size. **(B)** Average and standard error - across subjects - of the R^2^ derived for the last 10 epochs of the *BL* phase and the first and last 10 epochs of *Pert* phase using the *NMFFixedW* and *NMFFixedC* algorithms. Results are shown for the unilaterally (top plots) and the bilaterally (bottom plots) extracted synergies. The p-values of Friedman’s ANOVA tests (significance level α = 0.1) are presented in each plot. Minimum Significance Difference tests showed significant differences across pairs (see *Materials and Methods*) as highlighted by the * symbol and the horizontal lines.

We found that for both legs, during both the X and X_inv_ experiments, the shared subspace dimensionality between *BL* and *late*-*Pert* synergies remained at 3, regardless of whether 4, 5, or 6 synergies were extracted from the *late-Pert* EMG data, and whether unilateral or bilateral synergy subspaces were compared (Fig. 4A). Indeed, if there were, say, 1 additional synergy activated at the end of the *Pert* phase, one would expect the 4-synergy *BL* space and the 5-synergy *late*-*Pert* space to share a 4-D, instead of a 3-D, subspace. We concluded that motor adaptation during both the X and X_inv_ experiments did not occur by recruiting a new muscle synergy while preserving the *BL* muscle synergies. It should be emphasized that, in this type of analysis, the values of the cosine of the principal angles do not matter. What matters is whether the patterns shown in Fig. 4A change as we increase the number of synergies used to model the EMG data collected during the *late-Pert* phase.

Another argument that supports the exclusion of mechanism *a* comes from the analysis of the R^2^ of the EMG data reconstruction. If adaptation happened to be associated with the recruitment of an additional 5^th^ muscle synergy, reconstructing the EMG activity during the *late-Pert* phase using 4 synergies would result in a decrease in the quality of the reconstruction as reflected by smaller R^2^ values. We, on the other end, showed that 4 synergies could represent equally well muscular activities during both the *BL* phase and the *late*-*Pert* phase of the experiment (Fig. S2).

### No modification of the weights of baseline muscle synergies during motor adaptation

We then tested if adaptation is achieved via modification of the activation patterns of a fixed set of muscle synergies (mechanism *b*) or via modification of the weights of select muscles in one of the 4 *BL* synergies (mechanism *c*). To test these hypotheses we determined how well the EMG data of the *BL* and *Pert* phases could be reconstructed by the reference *BL* muscle synergies while fixing either the *BL* muscle-synergy vectors (i.e., the *W* matrix of the Non-negative Matrix Factorization -NMF) (*NMFFixedW*), or the *BL* activation patterns of the synergies (i.e., the *C* matrix of the NMF algorithm) (*NMFFixedC*), (see *Materials and Methods*). A significant decrease in reconstruction quality in one of these analyses would indicate that either the same reference *W* (for *NMFFixedW*), or the same reference *C* (for *NMFFixedC*), was not sufficient to capture EMG changes associated with adaptation, thus implicating mechanism *c* in the former, or mechanism *b* in the latter, assuming that either *W* or *C* would be primarily modified for adaptation.

The bilateral analysis was primarily employed to highlight if mechanisms b and c, if present, are employed independently by the two legs or in a bilaterally-linked way. In the bilateral analysis (Fig. 4B), we observed that fixing either the muscle synergies or the activation patterns to their reference values led to a decrease in the reconstruction quality from the *BL* to the *Pert* EMG data irrespective of whether we considered the beginning (*Early Pert*) or the end (*Late Pert*) portions of the *Pert* phase. Thus, if the muscles from both sides are analyzed together, it is not possible to differentiate whether mechanisms *b* or *c* may better characterize adaptation drives. Indeed, for bilateral synergies, changes in the EMG characteristics from the *BL* phase to the *Pert* phase could only be captured if both *W* and *C* were left free to be updated in the NMF algorithm (Fig. S2).

In the unilateral analysis, implementing *NMFFixedC*, but not *NMFFixedW*, led to a drop in the reconstruction quality from the *BL* to *Pert* EMG data in both legs of both perturbation experiments (Fig. 4B). Thus, in both the X and X_inv_ experiments, the *Pert* data could not be modeled by just modulating the weights of the *BL* muscle synergies while keeping the temporal activations constant. However, adaptation could be modelled by modulating the muscle synergy activation patterns while leaving the composition of the muscle synergies unchanged. These results are consistent with mechanism *b* (fixed muscle synergies) and not with mechanism *c* (altered synergies post-adaptation). Furthermore, the concurrent observation that mechanism *b* was independently present in the muscle synergies of both legs in our unilateral, but not bilateral, analysis suggests that adaptation to the two perturbations is driven by unilateral mechanisms. It is worth pointing out that both legs could display changes in the temporal activations of the muscle synergies that would however be leg-specific. This latter point explains the inconsistency between the bilateral and unilateral results, and, more specifically, why mechanism *b* is not observed in the bilateral analysis. In fact, our results in the unilateral analysis suggest that left and right synergies that are linked in the bilateral analysis adapt their activation patterns independently, with different behaviors and time constants (as better explained later). Thus, when the synergies are bilaterally linked in the analysis, the changes in the resulting activation pattern cannot capture the different dynamics of the unilateral synergies.

### The X and X_inv_ perturbations elicited two distinct behaviors in the synergy activations

**Figure 5:**
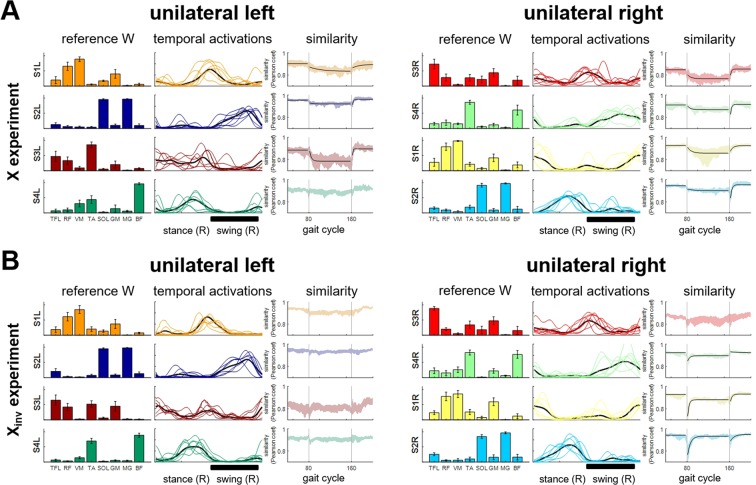
Adaptation in the unilateral activation patterns of the synergies. Results obtained using the *NMFFixedW* algorithm on all the EMG epochs in the two experiments, **(A)** for X and **(B)** for X_inv_. For each panel, each row shows the data for a different synergy (S#L for left side synergies, S#R for right side synergies). For each synergy, the plots (from left to right) show: (1) the average - across subjects - synergy reference modules; (2) the average - across epochs - temporal activation pattern during the last 10 gait cycles of *Pert* phase (thin colored lines), and the average - across segments and subjects - reference temporal activation pattern (bold black line), with a black bold line at the bottom of each set of plots representing the time interval when the force-field was active; and (3) the similarity (i.e., Pearson’s linear correlation coefficient) between the activation pattern of each epoch of the experiment and the reference activation pattern (that is the average of the epochs extracted during *BL*); the vertical grey lines represent the beginning and the end of the *Pert* phase. Black lines represent the exponential best fitting of the *Pert* and *AE* data. For the *BL* phase, the black lines represent the average similarity during *BL*. Exponential fitting was added only for the synergies for which it was possible to fit two exponential functions for the *Pert* and *AE* phases with a cumulative R^2^ > 0.75.

The above-summarized analyses indicate that step-length adaptation during the X and X_inv_ experiments can be modelled by modulating the temporal activations of the muscle synergies that mark unperturbed walking. We next focused on assessing how the activation of each synergy changed during the adaptation experiments. The dynamics of this change was evaluated by measuring the similarity between the temporal activation of 2-step epochs, during the *BL*, *Pert* and *AE* phases, and that of the corresponding reference synergy extracted from the whole *BL* data. This analysis resulted in similarity value time series (Fig. 5) charting the extent to which the synergy activations deviated from the reference patterns during the experiment. To determine if the activation of each muscle synergy displayed an adaptive behavior, we fit an exponential function (Eq. 3, *Materials and Methods*) to the corresponding similarity value time series derived for the *Pert* and *AE* phases, respectively. The behavior was defined to be adaptive if the cumulative R^2^ of this fit was >0.75.

Over the *Pert* phase of the X experiments, we observed both a modest step-wise (i.e., feedback) behavior and an adaptive (feedforward) behavior marked by a gradual change in the temporal activation similarity value time series. The adaptive behavior was detected in 3 out of 4 synergies on the left (unperturbed) side, and in all 4 synergies on the right (perturbed) side (Fig. 5A). The adaptive behavior was characterized by a decay in the similarity value time series until a stable value was reached after a few gait cycles. The time constants of this decay ranged from 9.6 (S3L) to 64.4 (S1L) gait cycles (Table S1). After removal of the perturbation, the similarity was gradually restored to its *BL* level. This *AE* phase presented time constants ranging from 2.3 (S3L) to 37.4 (S1L) gait cycles. When we examined the temporal activations of the synergies during *late Pert* (Figs. 5A and S3), we noticed that adaptation was largely driven by an increase in the activation amplitudes of S1L and S4R. Both were active during the portion of the gait cycle when the perturbation was produced by the robot (Fig. 5A, black bold line). Also, adaptation was associated with increased activations of S3L and S1R. It is worth noticing that the adaptation time constants of this bilaterally-linked synergy pair (S3L + S1R) were different, despite the similar modulation observed in *late*-*Pert* (Table S1 shows all the time constants and associated 95% confidence intervals), further suggesting that full adaptation is achieved via unilateral mechanisms.

In the X_inv_ experiment, we also observed both a step-wise (i.e., feedback) behavior and an adaptive (feedforward) behavior marked by a gradual change in the temporal activation similarity value time series. However, unlike the X experiment, the time series of the temporal activation similarity values for the muscle synergies of the right (perturbed) leg showed a dramatic change at the onset of *Pert* for several synergies. After this step-wise (i.e., feedback) behavior, the temporal activation similarity value time series showed a gradual return to a level close to the *BL* value. On the left (unperturbed) leg, even though S1L, S3L and S4L showed an initial step-like deviation from the *BL* value, the similarity value time series did not satisfy the set criterion of adaptation. On the perturbed right side, adaptation was visible in S1R, S2R and S4R. While all 3 synergies presented an abrupt degradation in similarity at the beginning of the *Pert* phase, S4R and S1R converged to levels more deviated from the average *BL* similarities at the end of *Pert* while S2R returned to its *BL* value. The time constants of adaptation ranged from 7.6 (S1R) to 15.8 (S2R) gait cycles in the *Pert* phase, and from 9.0 (S1R) to 21.2 gait cycles (S2R) in the *AE* phase (Table S1). Full adaptation to X_inv_ at the end of the *Pert* phase was characterized mainly by an increase in the activation of the synergies of the right leg (Fig. 5B). Changes in the temporal activations are shown in Fig. S3.

Finally, we analyzed if the two perturbations led to similar patterns of adaptation. We observed that, although the reference modules and activation patterns extracted during the *BL* phases of the two experiments were consistent, the activation patterns at the end of the *Pert* phase for the two perturbations were different (Fig. S4).

## Discussion

Our results show that the activity of lower-limb muscles during locomotor adaptation to the perturbations tested in the study can be modelled by modulating the muscle synergy activation patterns observed during the *BL* phase of the experiment (i.e., in the absence of any perturbation) while leaving the composition of the muscle synergies unchanged. Furthermore, the study provided evidence that the response to perturbations that cause locomotor adaptation is not associated with a single response strategy, but instead, by dynamically mixing multiple response mechanisms in a context-dependent manner. Specifically, our study unravels concurrent actions of unilateral feedforward- and feedback-driven mechanisms that affect the muscle synergies of the two legs and that are a function of the biomechanical effect of the perturbation. These results provide a clear demonstration of the invariance to mechanical perturbations of the human locomotor muscle synergies and show that their temporal activations are under both feedforward and feedback control in a context-dependent fashion.

### Separating feedforward- and feedback-driven EMG changes during locomotor adaptation

Motor behaviors associated with feedback (i.e., reactive) and feedforward (i.e., adaptive) mechanisms were previously observed during adaptation experiments relying on applying unilateral resistive perturbations to the hip and knee^21^, moving platforms acting on the stance leg^22^, split-belt treadmill^16,20,24^ and asymmetric cycling^23^. Feedforward-controlled behaviors are marked by error-driven, progressive modifications of motor commands - based on an internal model of the perturbation - that converge toward an adapted state. They are associated with an aftereffect once the perturbation is removed^14,16^. This type of control has been attributed to the cerebellum, whose role in updating the internal representation of the dynamics of the limb through sensory prediction error has been observed in both the upper^34,35^ and lower limbs^24,36^. On the other hand, motor behaviors that are the result of purely feedback mechanisms are marked by abrupt changes in response to the perturbation and are not associated with an aftereffect when the perturbation is removed. Feedback mechanisms are primarily driven by spinal circuits^37^.

**Figure 6:**
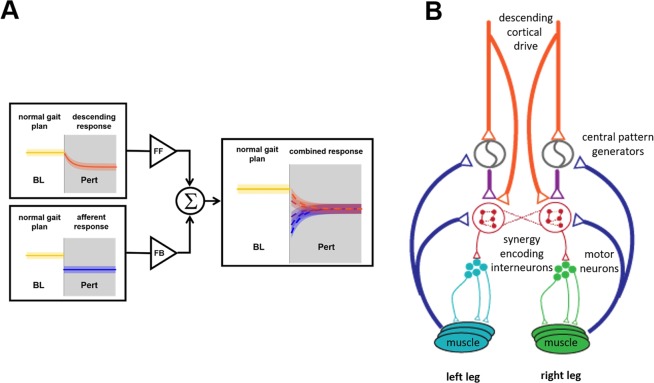
Theoretical and physiological model of the neuromuscular adaptation process as a sum of feedback and feedforward components. The adaptation behaviors that we observed in the similarity plots of Fig. 6 can be explained as the weighted sum of an exponential feedforward (FF) adaptation and a step-like feedback response (FB) **(A)**. By modifying the relative weights of these two components, it is possible to explain all the behaviors we observed in the muscle synergies (a characterization of this model and its fitting on our data are presented in Figs. S9 and S10). At the physiological level **(B)** our results suggest a direct or indirect (through the rhythm generation parts of the CPGs) activity of both descending corticospinal drive and muscular afferents on the networks of interneurons encoding the muscle synergies in the spinal cord.

The presence of feedforward and feedback adaptations in the EMGs has been investigated in the past by examining, in a setup similar to ours, the presence or absence of changes in the muscular activations during catch trials interspersed in the perturbed conditions^21^ or, more recently in the split-belt treadmill experiment, by comparing the aftereffect of a long exposure to the perturbation with the initial response to a perturbation of opposite direction^20^. Here, we show that these components of adaptation can also be characterized at the muscle-synergy level, and that this analysis provides valuable information on how groups of muscles adapt and/or react in concert during prolonged perturbations. The synergy activation changes that we observed during both the X and X_inv_ experiments can, in fact, be explained as the summation of feedback- and feedforward-driven responses (Figs. 6A, S9 and S10). The feedback-driven component observed at the onset of the *Pert* phase of the experiments is triggered by the initial deviation from the gait plan that marks the *BL* phase. This component accounts for the abrupt changes observed at the beginning of the *Pert* phase. Such step-like responses were observed in the X experiment (e.g., S1L and S2R, Figs. 5A and S10) but were particularly prominent in the X_inv_ perturbation (S1R, S2R and S4R, Figs. 5B and S10). The feedforward response, on the other hand, drives the adaptation from the initial feedback-mediated response towards the final adapted state and is marked by an exponential time-course.

By analyzing the muscle synergies during the adaptation process, we successfully disentangled the feedback-driven EMG changes from those mediated by feedforward descending drives. Importantly, this separation allowed us to determine the role of the different feedback and feedforward mechanisms, operating on distinct muscle synergies with different time-courses, in response to different perturbations. The results also show how feedback and feedforward mechanisms affect simultaneously the activation patterns of multiple lower-limb muscles. The results we found on the presence of feedback and feedforward components of adaptation for the X_inv_ are consistent with those found by Lam and colleagues on a similar setup^21^.

### Context-dependent locomotor adaptation

Previous studies have provided conflicting results in regard to whether locomotor adaptation is driven by unilateral or bilateral control. For instance, Choi and Bastian^12^ provided evidence of independent high-level neural control of the two legs, whereas Houldin *et al*.^38^ showed inter-limb transfer of learning during force-field adaptation, and Maclellan *et al*.^25^ suggested that bilaterally-linked unilateral modulations of CPG activities of the two legs takes place during split-belt adaptation.

The results of our study show that the response to perturbations that cause locomotor adaptation is context-dependent. Analysis of the results of the X experiment showed a modest feedback response and a dominant feedforward response in both legs, though the response observed in the data recorded from the right (perturbed) leg followed a different time-course of neuromuscular adjustment from the left (unperturbed) leg. In contrast, the results of the X_inv_ experiment showed a predominantly unilateral response affecting the right (perturbed) leg. The data collected from the left (unperturbed) leg showed only modest changes consistent with small feedback adjustments. The data from the right (perturbed) leg showed large feedback adjustments and a clear adaptation response in three out of four muscle synergies. Taken together, these results show that the relative contributions to locomotor adaptation of the two legs are context-dependent.

But why did we observe different contributions of feedback- and feedforward-mediated responses to the two perturbations? In previous work^19^, we showed that the primary task-relevant factor driving the response to the X and X_inv_ perturbations is the need for preserving long-term gait stability. As both perturbations triggered a response to preserve the same long-term gait stability plan, one would think that the discrepancy in strategy that we observed at the neuromuscular level in response to the two perturbations is due to the different effects the perturbations have on the subject’s dynamic stability.

Specifically, we argue that the larger feedback component observed during the X_inv_ vs. the X experiment is likely due to the more immediate balance threat induced by the X_inv_ perturbation compared to the X perturbation. The X_inv_ perturbation causes a mechanical effect that is similar to hitting an obstacle during the swing phase of the gait cycle. This is expected to bring the projection of the center of mass close to its stability boundaries in the antero-posterior direction. Hence, it is not surprising that we observed a change in the activations of the knee extensors during swing (S1R) and of the ankle plantar-flexor muscles during stance (S2R) - a response that is modulated over time to achieve an increase in the activation of the synergies (Fig. S3). In fact, previous studies have shown short- and medium-latency reflex responses in multiple muscles that appeared to be mediated by Ia and II afferents, respectively, likely caused by the overall jar that the stumbling causes on the perturbed leg^39^.

Unlike the X_inv_ perturbation, the X perturbation has an effect that is similar to delaying foot landing. A study by van der Linden *et al*.^40^ showed that unexpected delayed foot landing triggers a reflex response on multiple muscles after heel-strike, consistent with the activation adjustment we found in synergy S2R. Nevertheless, in our experiment the sensory feedback provided by the X perturbation acting on the leg likely prompted the subject to anticipate the forthcoming missteps, thus making the effects of the feedforward control to be dominant over those of the feedback response. We argue that, at the biomechanical level, the effects of the X perturbation are less immediately threatening the control of balance, but may lead, over time, to an unstable gait pattern, thus triggering a feedforward adjustment of the motor plan^19^.

All in all, our results demonstrate that the neural strategy underlying the response to perturbations that cause locomotor adaptation is context-dependent^12^. Interestingly, the complexity of the mechanisms underlying the response to these perturbations is not apparent when one examines simple aspects of the biomechanics of gait^19^, but it is clearly shown by examining the characteristics of the muscle synergies derived from the EMG data collected during the experiments^23^.

### The same muscle synergies modulated by feedback- and feedforward-based mechanisms

In contrast to the changes in the activation of muscle synergies, changes in the EMG data of individual muscles in response to the perturbations were more subtle (Fig. S5). In fact, similarity plots derived from data recorded from individual muscles showed adaptation (though not as clearly as the muscle synergies) only in a few cases (Figs. S6 and S7).

Our results provide strong evidence that in humans, both descending (feedforward) and afferent (feedback) drives project, either directly or indirectly, onto the same spinal interneuronal networks that encode locomotor muscle synergies (Fig. 6B). While a modular organization of the control of muscles has been demonstrated for descending motor signals in both animal models^41,42^ and humans^9,25,26,30^, the mechanisms controlling synergistic reflexes are still poorly understood. It is known that several reflex pathways project to second and third order interneurons and are able to modulate the activation of the CPGs^43^. Recent studies on animal models have hypothesized a two-level organization of the CPGs^44,45^, with the upper-level neural oscillators commanding the lower-level pattern formation interneuronal networks.

In a previous rodent optogenetic study, Levine and colleagues^28^ identified a molecularly defined population of spinal interneurons that encode the coordination of multiple muscles and are simultaneously activated by both cortical and afferent drives. These networks appear to be the building blocks enabling complex feedforward- and feedback-driven behaviors and could correspond to the hypothesized pattern-formation networks at the lower level of the CPG. In humans, the possibility that sensory afferents could directly act on muscle synergies has been suggested^26,30,31,46^ but shown only by Chvatal and Ting^22^, who observed anticipatory and reactive modulations of muscle synergies in response to single-step platform perturbations of the stance leg. The results herein presented extend such observations to motor adaptation and provide further evidence that sensory afferents have a direct effect on muscle synergies by altering their recruitment together with efferent commands during continuous exposure to forces.

As a final remark, the results of the study show that the neuromuscular response of a user to the forces exerted by an exoskeleton during gait does not necessarily lead to a modification of the descending drive to the muscles, which is a primary goal of robot-assisted gait training. Being able to discern whether the neuromuscular responses to the robot-induced forces are reactive or proactive could provide the foundations for new approaches to the design of robot-assisted gait rehabilitation procedures^29,47^. This could be done, for example, by designing assistive or perturbing forces that trigger descending responses in the activation of selected synergies. Another, similar, approach could be based on analyzing in quasi-real time the neuromuscular adaptation to the forces that the robot is administering during the therapy and use this information for tuning the robotic assistance so as to promote proactive responses or curb reactive ones.

## Materials and Methods

### Study design

Nine healthy adults (3 women; age = 27.8 ± 3.5; height = 177.7 ± 6.9 cm; mass = 72.4 ± 10.6 kg) participated in the study. This was a sample of convenience. Exclusion criteria for the study were the presence of orthopedic or neurological conditions with a potential effect on the performance or outcome of the experiments. Three of the subjects had experienced the motor perturbations used in the study in a previous experiment. All data collections were performed at Spaulding Rehabilitation Hospital, Boston MA. Subjects completed all the experimental procedures in a single day. The experimental protocol was approved by the Spaulding Rehabilitation Hospital Institutional Review Board. Subjects signed an informed consent form prior to participating in the study. All study procedures were carried out in accordance with all relevant guidelines and regulations.

### Generating perturbations using a robotic system

#### Device settings

Subjects walked with the lower limbs strapped to the robotic legs of a system designed for use in robot-assisted gait therapy (Lokomat by Hocoma AG, Switzerland)^48^. The robotic system (Fig. 1A) allows for both flexion and extension control of the hip and knee joints via linear actuators. Potentiometers are used to track the joint angles at the hip and knee. Force transducers are used to monitor the torques generated at the joints. The Path Control^49^ was used in the study. This control modality allowed subjects to naturally control the timing of their gait. The controller estimated, at every instant, the position in the gait cycle via comparison of the actual hip and knee joint angular displacement and angular velocity with template patterns. The comparison used a norm-distance minimization algorithm^50^. The Generalized Elasticities method^51^ was implemented to make the system as transparent as possible to the subjects during walking. The Body Weight Support capability of the system was not utilized in the study.

#### Perturbations

During the experiments, the robot was used in one of two modes of operations: Back-Driven Mode (BDM) and Force Field Mode (FFM) (Fig. 1D). In the BDM, the robot’s apparent impedance was set to zero to minimize the effect of the robot on the walking patterns (i.e., the system was as “transparent” as possible to the subjects). In the FFM, a perturbation was applied to the right leg during the swing phase of the gait cycle. The perturbation force was generated by the robot according to the following equation:

1 $${\rm{F}}({\rm{t}})=[\begin{array}{c}{{\rm{F}}}_{{\rm{x}}}({\rm{t}})\\ {{\rm{F}}}_{{\rm{y}}}({\rm{t}})\end{array}]=[\begin{array}{cc}{\rm{A}} & 0\\ {\rm{B}} & 0\end{array}][\begin{array}{c}{{\rm{v}}}_{{\rm{x}}}({\rm{t}})\\ {{\rm{v}}}_{{\rm{y}}}({\rm{t}})\end{array}]$$

where *F*_*x*_ and *F*_*y*_ represent the antero-posterior (*x)* and vertical (*y*) components of the perturbing force acting on the foot, and *V*_*x*_ and *V*_*y*_ represent the *x*- and *y*-components of the foot velocity as reconstructed using the following Jacobian:

2 $$J(\theta )=[\begin{array}{ll}{l}_{Femur}\,\cos ({\theta }_{h})\,+\,{l}_{tibia}\,\cos ({\theta }_{h}-{\theta }_{k}) & \,-\,{l}_{tibia}\,\cos ({\theta }_{h}-{\theta }_{k})\\ {l}_{Femur}\,\sin ({\theta }_{h})\,+\,{l}_{tibia}\,\sin ({\theta }_{h}-{\theta }_{k}) & \,-\,{l}_{tibia}\,\sin ({\theta }_{h}-{\theta }_{k})\end{array}]$$

where *l*_*Femur*_ is the length of the femur, *l*_*Tibia*_ is the length of the tibia, and *θ* is the vector of the joint angles *θ*_*h*_ at the hip and *θ*_*k*_ at the knee.

Subjects experienced two different perturbations produced by different values of the gains *A* and *B* shown in Eq. 1. The first perturbation, denoted by X, was achieved by setting $A=0.115\,\left(\frac{N\cdot s}{Kg\cdot m}\right)\cdot SubjMass$ and *B* = 0, where *SubjMass* is the subject’s body mass in Kg. The second perturbation, denoted by X_inv_, was achieved by setting $A=0.092\,\left(\frac{N\cdot s}{Kg\cdot m}\right)\cdot SubjMass$ and $B=-0.058\,\left(\frac{N\cdot s}{Kg\cdot m}\right)\cdot SubjMass$. These values of *A* and *B* were previously determined as suitable to induce modifications in step length (~30% of baseline length) in the forward (X perturbation) and backward (X_inv_ perturbation) directions, respectively, without concurrently affecting step height^19^.

### Study procedures

Footswitches were attached to the soles of the subject’s shoes in positions corresponding to the calcaneous bone and the first metatarsophalangeal joint, respectively. Subjects were strapped to the system using the cuffs of the robotic legs. Then surface electromyographic (EMG) electrodes were placed on the following 16 muscles (8 per leg): tensor fascia latae (TFL), vastus medialis (VM), rectus femoris (RF), biceps femoris (BF), medial head of the gastroecnemius (MG), tibialis anterior (TA), soleus (SOL) and gluteus maximus (GM). Electrodes were positioned as close as possible to the cusp of the muscle belly following the SENIAM guidelines^52^ but avoiding contact with the cuffs of the robotic legs, which would have caused artifacts in the EMG recordings (Fig. 1B). The EMG and footswitch signals were digitized using 12 bits with a sampling rate of 3 kHz. Data collected from the robotic system was synchronously recorded at 1 kHz.

Six of the nine subjects who participated in the study had no previous experience with walking using the robotic system. In these subjects, we performed a 5-minute habituation trial using the system in BDM.

#### Motor adaptation paradigm

During all the experimental trials, the treadmill speed was set at 3 km/h. Subjects’ cadence was paced using a metronome set at 84 beats per minute. Subjects were instructed to pace their heel strikes with the beats of the metronome. No further instructions were given to the subjects. The metronome was used, as in our previous work^19^, to stabilize the pace, and consequently the speed of the foot. This was done to make sure that subjects experienced consistent perturbation magnitudes across different steps. We have shown in our previous work that the metronome does affect the adaptation process^19^.

A total of 4 trials were performed as follows: (1) a 90-s trial, for estimating the parameters of the Generalized Elasticities method; (2) a 120-s trial performed with the optimized controller, for estimating the subject’s baseline walking pattern that was used as a reference during the experiment; (3) a 10-min trial to test the effects of the X (or X_inv_) perturbation; and (4) a 10-min trial to test the effects of the X_inv_ (or X) perturbation experiment. The order in which the X and X_inv_ perturbations were tested was randomized across subjects. A 5-minute break was allowed between the X and X_inv_ experiments. In each of the X or X_inv_ trials, subjects performed 420 gait cycles. The trials consisted of four phases.

(i) The habituation phase consisting of 180 gait cycles during which the system was set in BDM. 9 gait cycles during which the system was set in FFM were randomly selected after the first 40 cycles, so that there were at least 8 and no more than 18 BDM gait cycles between any two FFM gait cycles. The FFM gait cycles, named *single step perturbations*, were used to estimate each subject’s response to a single-cycle perturbation.
(ii) The baseline phase (*BL*) consisting of 80 consecutive BDM gait cycles.
(iii) The perturbation phase (*Pert*) consisting of 80 consecutive FFM gait cycles.
(iv) The aftereffect phase (*AE*) consisting of 80 consecutive BDM gait cycles.

### Biomechanical analysis of adaptation

Step length was defined, consistently with our previous work^19^, as the foremost position of the foot during swing. It is important to notice that, in our set up, the reference coordinate system used to determine step length is aligned with the center of rotation of the hip. Hence, if foot contact had occurred with the ankle aligned with the hip, the estimated step length would have been equal to zero. For each subject, the step length values were normalized to the average step length recorded during the *BL* phase. After normalization, for each of the X and X_inv_ experiments, the data was averaged across subjects to determine the aggregate adaptation behavior. The time series of the step length estimates derived from the data collected during the *Pert* and *AE* phases of the experiment were modelled using exponential functions:

3 $$f(step)=\alpha \cdot ex{p}^{\beta \cdot step}+\gamma $$

where *α* represents the response to the perturbation (for the *Pert* phase) or removal of the perturbation (for the *AE* phase) observed at the beginning of the *Pert* or *AE* phases, *β* is the time constant of the adaptation, and *γ* the asymptotic step length value observed when full adaptation has been achieved. In this analysis, similarly to what we did in previous work^19^, *α* was estimated by averaging - across all subjects - the step length values observed during the 9 *single step perturbations* during the habituation phase of the experiment, and *β* and *γ* were estimated using a least-squares algorithm.

### EMG Pre-processing and segmentation

The EMG data was visually inspected to ensure that high-quality data was then processed. The trials and channels showing significant movement artifacts were excluded from the analysis. This process led to selecting data from 7 out of 9 subjects who participated in the study. The EMG data was first band-pass filtered (50 to 450 Hz) using a 7^th^-order elliptic filter. The filtered signals were then full-wave rectified and low-pass filtered (cut-off of 5 Hz) using a 7^th^-order elliptic filter, thus deriving the EMG envelopes. The heel-strike and toe-off times were extracted from the footswitch data of each foot. These events were used to segment the EMG data so that each segment corresponded to 2 steps. The length of each segment was normalized, and the data was re-sampled so that each segment consisted of 200 data points. The amplitude of the EMG data collected during all experimental phases was normalized to the median, across all *BL* segments, of the maximum values derived for each segment.

### EMG analysis

#### Extraction of the muscle synergies

The non-negative matrix factorization (NMF) algorithm was used to estimate the muscle synergies from the normalized EMG envelopes. This algorithm models the muscular activity *MA* as a linear combination of muscle synergies, *W*, whose activity is modulated over time by a set of activation patterns, *C*:

4 $$MA(t)=\mathop{\sum }\limits_{i=1}^{N}{C}_{i}(t){W}_{i}$$

To derive *W* and *C*, we used the NMF algorithm, which is based on the assumption that the EMG envelopes are corrupted by an uncorrelated Gaussian noise^53^. Muscle synergies were extracted unilaterally (i.e., the EMG data collected from the left leg and the right leg was analyzed separately) as well as bilaterally (i.e., the data collected using all 16 channels was analyzed together). For the *BL*, *Pert* and *AE* phases of each experiment and each subject, we extracted synergies from each 2-step epoch so that a total of (420 − 180)/2 = 120 synergy sets were obtained. A validation of this approach to extracting muscle synergies is shown in Fig. S8. In addition, reference muscle synergies (*REF*_*synX*_ for the X experiment, and *REF*_*synXinv*_ for the X_inv_ experiment) were extracted, unilaterally as well as bilaterally, from the whole *BL* data (80 gait cycles) of both experiments without data segmentation. Subsequently, an average activation pattern for the reference synergies was calculated by averaging - across step cycles - the reference *C*, segmented in time before averaging using the same method utilized to segment the EMG envelopes.

#### Number of muscle synergies

The R^2^ derived using the reconstructed EMG data (based on the muscle synergies) and the original EMG data was utilized to select the number of synergies suitable to model the EMG data. Specifically, we selected the number of muscle synergies that would yield a R^2^ > 0.75 both in the unilateral and bilateral analyses for all subjects. This criterion was met by selecting 4 synergies (R^2^ = 0.80 ± 0.02 for the bilateral analysis; R^2^ = 0.86 ± 0.06 for the unilateral analysis of the left leg EMG data; R^2^ = 0.79 ± 0.04 for the unilateral analysis of the right leg EMG data). This result is consistent with previous studies^8^.

#### Changes in muscle synergies in response to the perturbation

To investigate the possible recruitment of additional synergies during motor adaptation, we also extracted 5 unilateral synergies and 5 and 6 bilateral synergies (Figs. 4A,B and S2). We then quantified the similarity between the subspaces spanned by the reference *W* and the epochal *W* across the experimental phases. The reference *W* was obtained by using all the segments of data collected during the *BL* phase. The epochal *W* was derived for each segment of the data collected during all experimental phases. The cosine of the principal angles^54^ between the subspaces spanned by the muscle-synergy sets was then derived to quantify the similarity between the above-defined subspaces. High values of the cosines of the principal angles (i.e., close to 1) indicate that the subspaces spanned by the two synergy sets intersect over a shared subspace of *n* dimensions, where *n* is the number of cosine values above threshold. The threshold value was derived - as previously proposed by Cheung *et al*.^55^ – via the following surrogate analysis. We calculated, for each subject, the cosines of the principal angles between the reference synergy set (*REF*_*synX*_ or *REF*_*synXinv*_) and 1,000 Gaussian noise-corrupted (μ = 0; σ = 0.1) versions of each of the 40 *BL* (two-step) segments. For each of these segments, we estimated the 95^th^ percentile value of the distribution of the cosines of the principal angles obtained for each of the 1,000 Gaussian noise-corrupted versions of the *BL* segments. Finally, the median of this percentile value across the 40 *BL* epochs was computed and selected to be the threshold for determining the dimensionality of the shared subspace *n* for each subject. This procedure allowed us to account for the variability of the epochal muscle synergies expected from chance when determining the threshold.

We also extracted *C* or *W* from every epoch by either fitting fixed *BL* synergies, *W* (*NMFFixedW*), or fixed *BL* activation patterns, *C* (*NMFFixedC*), to the epoch data. Through all iterations of the NMF algorithm, the *W* (for *NMFFixedW*) or *C* (for *NMFFixedC*) matrix was fixed at its reference value, thus allowing for updating only the *C* (for *NMFFixedW*) or *W* (for *NMFFixedC*) matrix. Using this approach, the variability of the original data set was described only by the updated parameter (i.e., *C* if *W* was fixed or *W* if *C* was fixed).

How the muscle-synergy activation coefficients *C* evolved over the course of adaptation was characterized by evaluating the similarity between *C* of each epoch and the reference *C* obtained from the *BL* data, quantified using the Pearson’s linear correlation coefficient. For each epoch, a similarity value was obtained, thus resulting in a time series of similarity values across the *BL*, *Pert* and *AE* phases. To facilitate subsequent modeling of the adaptation dynamics, this time series of similarity values was smoothed using an 8-point moving average filter, that was applied separately to the values from the three phases of the experiments. We fitted the exponential function in Eq. 3 to the similarity value time-series derived for the *Pert* and *AE* phases. All three parameters α, β and γ were estimated using a least-squares algorithm. The behavior was defined to be adaptive if the cumulative R^2^ was >0.75.

We analyzed the data to identify changes in muscle synergies between the two experiments. In this analysis we calculated the similarity between the average weights for the EMG data collected during the *BL* phase of the X and X_inv_ experiments. We also calculated the similarity between the temporal activation patterns during the *BL* and the *late-Pert* phases across the two experiments. The similarity between the weights was calculated using the normalized dot product. The similarity between the temporal activations was calculated using Pearson coefficients.

### Statistical analysis

Several statistical analyses were performed on the data recorded during the experiments. A statistical analysis was performed to test for significant differences in step length on the perturbed side during the different phases of the experiment (Fig. S1). We compared the average normalized step length during the *BL* phase and the step length for the first and last gait cycles of *Pert* and *AE* phases. The analysis was based on Friedman’s ANOVA test (p-values are presented in Fig. S1). The Minimum Significance Difference (MSD)^56^ test was used for pairwise comparisons of the values of step length between specific phases of the experiments. Similarly to what we did in^19^, we compared: (1) average step-length during late (last 5 cycles) *BL* versus first step of *Pert*, to test if the perturbation induced a significant change; (2) step-length values for the first step versus the last step of *Pert*, to test if subjects showed an adaptation to the perturbation force vector; (3) step-length values for late *BL* versus last step of *Pert*, to test if subjects fully compensated for the robot-induced changes in step-length, (4) step-length values for late *BL* versus first step of *AE*, to test for the presence of a significant aftereffect; (5) step-length values for the first step of *Pert* versus the first step of *AE*, to test if the aftereffect mirrored, in magnitude, the robot-induced change in step length; (6) step-length values for the first step versus the last step of *AE*, to test if subjects demonstrated changes in step length during this phase; and (7) step-length values for late *BL* versus the last step of *AE*, to test if subjects returned to baseline values of step length at the end of the aftereffect phase. The z-value for the MSD test was set to obtain an overall α of 0.05 for the seven comparisons considered, for an effective error rate per comparison equal to 0.0012^56^.

We extracted synergies (unilaterally and bilaterally) by either using the standard NMF algorithm, or by fixing *W* or *C* (Eq. 3) to their baseline values. The R^2^ values extracted in the different phases of both experiments were statistically compared for all these analyses using a Friedman ANOVA test (α = 0.1). Specifically, we compared values of R^2^ obtained unilaterally and bilaterally using the unconstrained and constrained NMFs between the *BL*, early *Pert* and *late-Pert* phases. The resultant p-values for these analyses are presented in Figs. S2 and 4B. The Minimum Significance Difference test was used to perform pairwise comparisons. The z-value for the MSD test was set to obtain an overall α of 0.05 for the three comparisons considered (baseline vs. early adaptation, baseline vs. late adaptation and early adaptation vs. late adaptation), for an effective error rate per comparison equal to 0.0083^56^. Finally, a statistical analysis based on the Wilcoxon’s signed rank test (α = 0.05) was used to test for statistically significant changes in the similarity of the activation patterns observed during the *BL* and *late-Pert* phases (Fig. S04).

## Supplementary information

Supplementary Materials (PDF).

## Data Availability

The data collected in the study is available on PhysioNet https://physionet.org/.

## Code availability

The code developed for the analysis of data collected during the study is available upon request to be sent to the corresponding author.
