# Supplementary material for: Robot-Driven Locomotor Perturbations Reveal Synergy-Mediated, Context-Dependent Feedforward and Feedback Mechanisms of Adaptation
Source: Sci Rep. 2020 Mar 25;10:5104. doi: 10.1038/s41598-020-61231-8 (PMC7096445; doi:10.1038/s41598-020-61231-8)
Supplement: Supplementary file 1 — Supplementary Materials (PDF). [file 41598_2020_61231_MOESM1_ESM.pdf]

Supplementary Materials

ROBOT-DRIVEN LOCOMOTOR PERTURBATIONS REVEAL SYNERGY-MEDIATED, CONTEXT-DEPENDENT FEEDFORWARD AND FEEDBACK MECHANISMS OF ADAPTATION

Giacomo Severini[1,2,3], Alexander Koenig[1], Catherine Adans-Dester[1], Iahn Cajigas[1,4], Vincent CK Cheung[5], Paolo Bonato[1,6]

[1] Dept of Physical Medicine & Rehabilitation, Harvard Medical School, Spaulding Rehabilitation Hospital, Boston MA, USA.
[2] School of Electrical and Electronic Engineering, University College Dublin, Dublin, Ireland.
[3] Centre for Biomedical Engineering, University College Dublin, Dublin, Ireland.
[4] Miller School of Medicine, University of Miami, Miami, Florida.
[5] School of Biomedical Sciences, and The Gerald Choa Neuroscience Centre, The Chinese University of Hong Kong, Hong Kong, China.
[6] Wyss Institute for Biologically Inspired Engineering, Harvard University, Boston MA, USA.

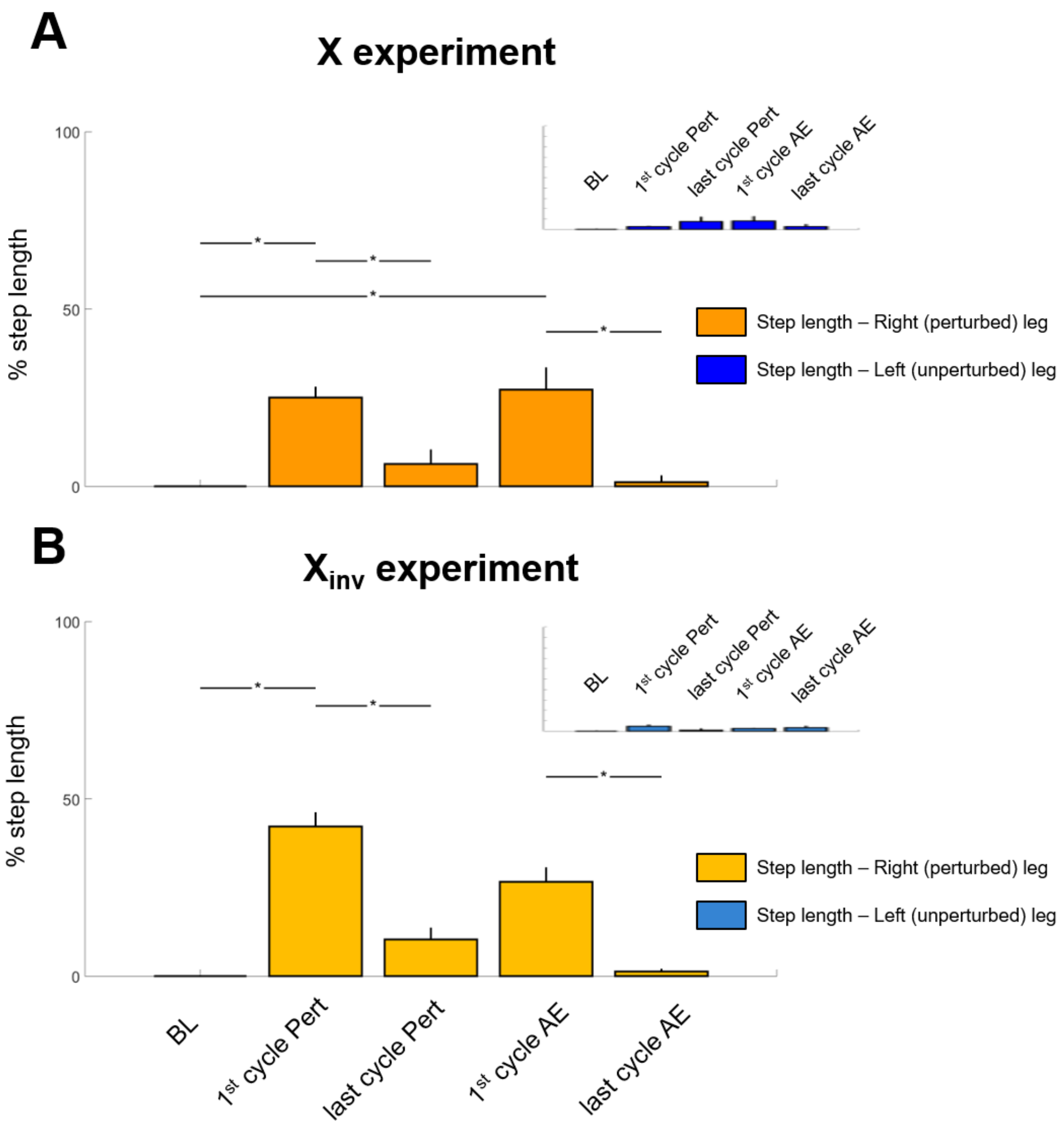

***Figure S1*. Aggregate statistical analysis of the changes in step length observed during the X and X_{inv} experiments.** Changes in step length for the right (larger plot) and the left (inset) lower limbs for the **(A)** X and **(B)** X_{inv} experiments. The right lower limb shows significant motor adaptation in step length in both experiments. The left lower limb does not show changes in step length reflecting motor adaptation in both experiments. * indicates significant changes among conditions estimated using Friedman ANOVA tests (p-value presented in the figure) followed by pairwise comparisons using the Minimum Significant Difference (MSD) test (see *Materials and Methods* for additional details). Data is shown for: baseline (*BL*), first gait cycle during the perturbation phase (1st cycle *Pert*), last gait cycle during the perturbation phase (last cycle *Pert*), first gait cycle during the aftereffect phase (1st cycle *AE*), and last gait cycle during the aftereffect phase (last cycle *AE*).

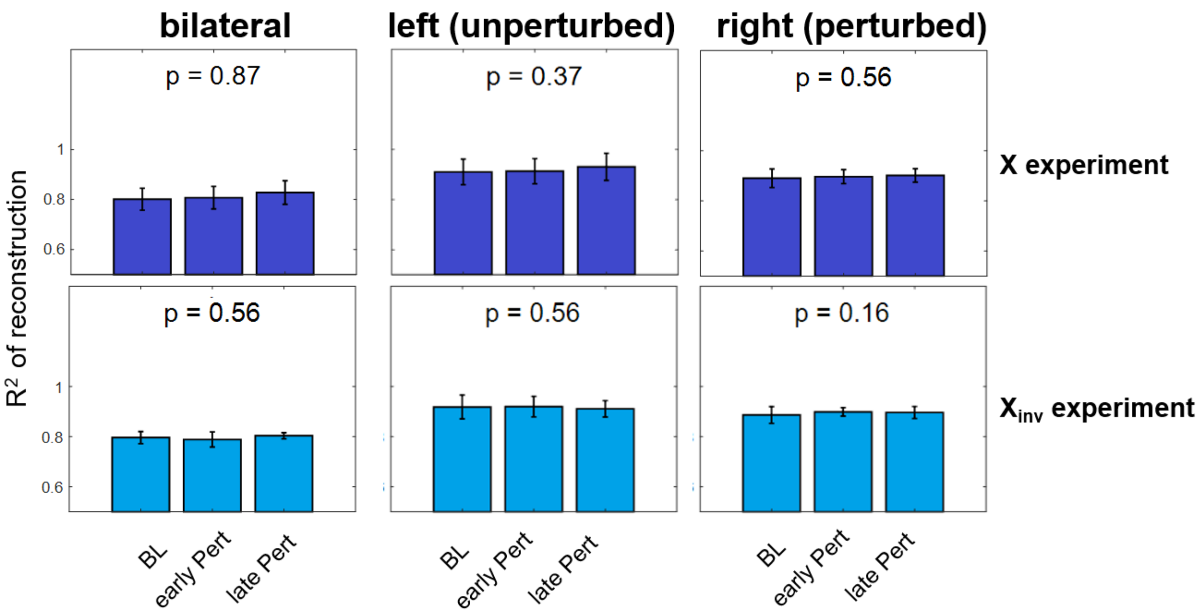

***Figure S2*. Changes in R2 of reconstruction using the standard NMF algorithm.** The bars represent the average and standard error (across subjects) of the quality of reconstruction for the data corresponding to the last 10 segments (i.e. 20 gait cycles) of the *BL* phase and the first and last 10 segments of the *Pert* phase using the standard unconstrained NMF algorithm. Dark blue bars represent the results obtained for the X experiment (top plots). The light blue bars represent the results obtained for the X_{inv} experiment (bottom plots). Statistical analysis based on Friedman's ANOVA was performed for each analysis and the relative p-values are presented in each plot. A post-hoc analysis based on the Minimum Significance Distance test was performed (see *Materials and Methods* for additional details). No statistically significant differences were observed.

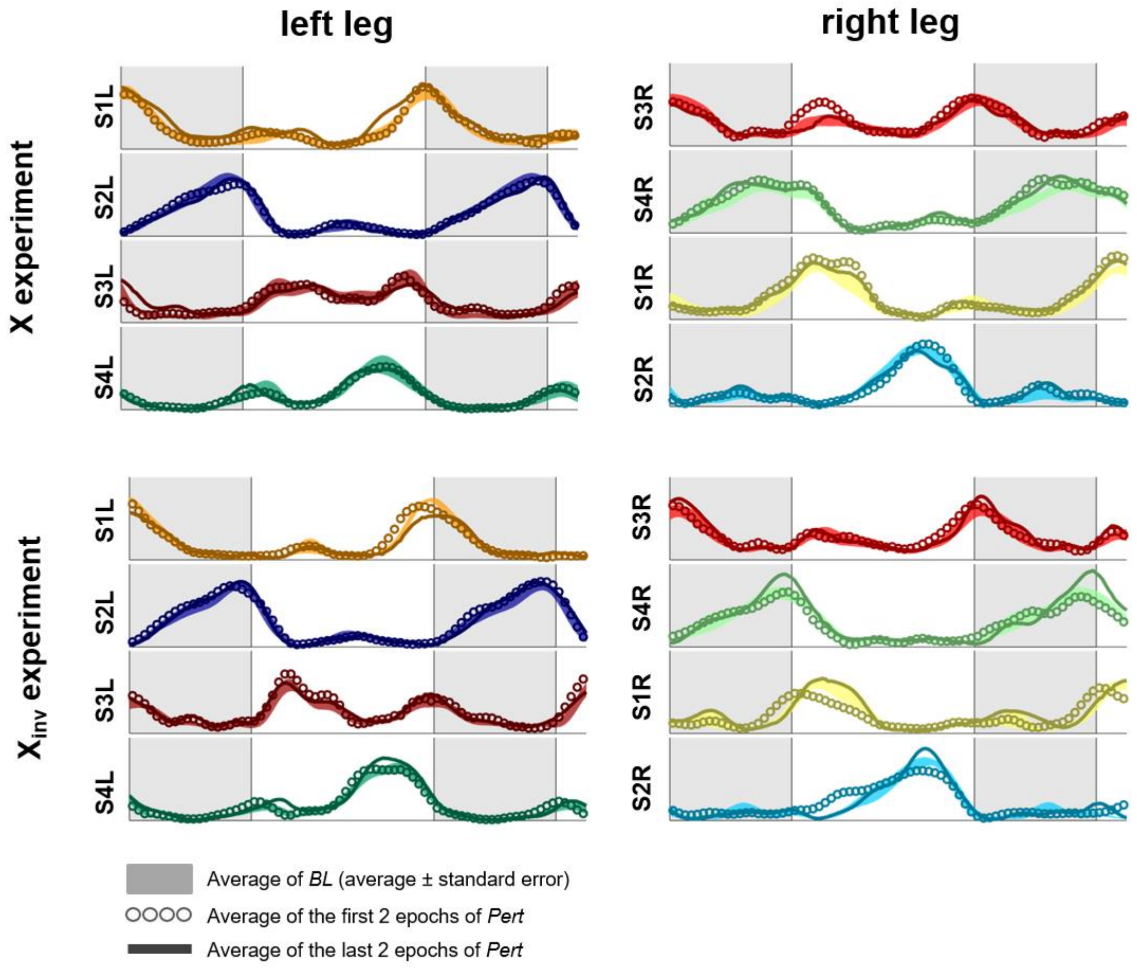

***Figure S3*. Activation coefficients for the *BL*, early *Pert* and *late-Pert* phases.** For each synergy, the trajectory represented by a shaded area (in different colors) corresponds to the average and standard error (across subjects) of the activation pattern observed during the *BL* phase. The dotted line (i.e., trajectory of open circles) represents the average (across subjects) activation pattern during the first 2 epochs (i.e., 4 gait cycles) of the *Pert* phase, while the solid dark line represents the average activation pattern during the last 2 epochs (i.e., 4 gait cycles) of the *Pert* phase. The grey shaded areas mark the phases of the gait cycle when the perturbation was active.

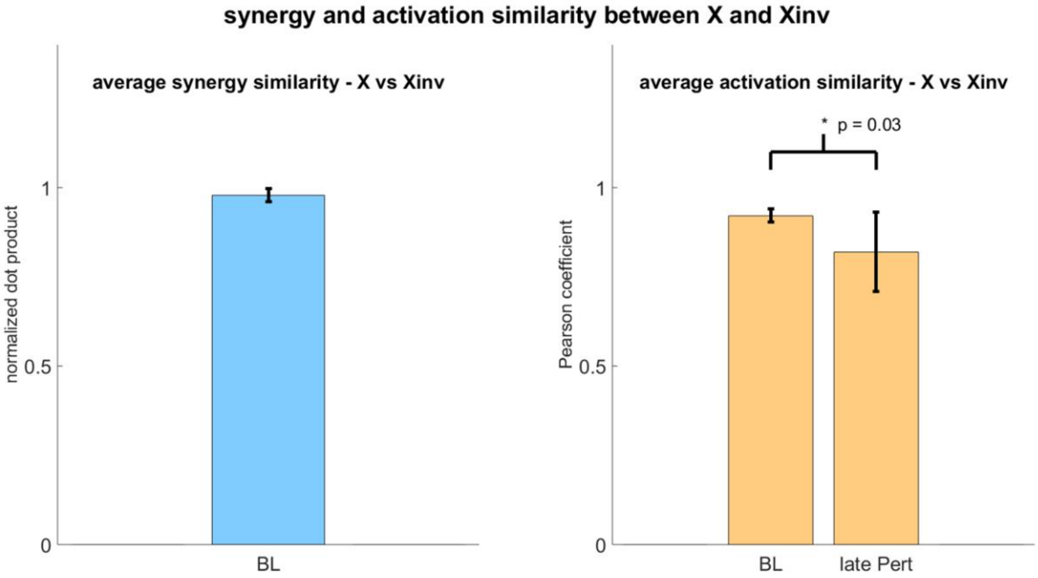

***Figure S4*. Similarity of the synergy modules and activation patterns between the X and X_{inv} experiments.** The plot on the left shows the average (across subjects) and standard error of the similarity between the *BL* synergy modules derived for the X and X_{inv} experiments. The similarity was calculated using the normalized dot product. The plot on the right shows the similarity between the activation patterns observed during the *BL* phase and the *late-Pert* phase for the X and X_{inv} experiments. The similarity was calculated using the Pearson correlation coefficient. We identified a significant decrease in similarity of the activation patterns ($p = 0.031$, Wilcoxon signed rank test) between the two experiments when comparing data collected during the *BL* phase and the *late-Pert* phase. The result indicates that the two experiments elicit two different adapted states.

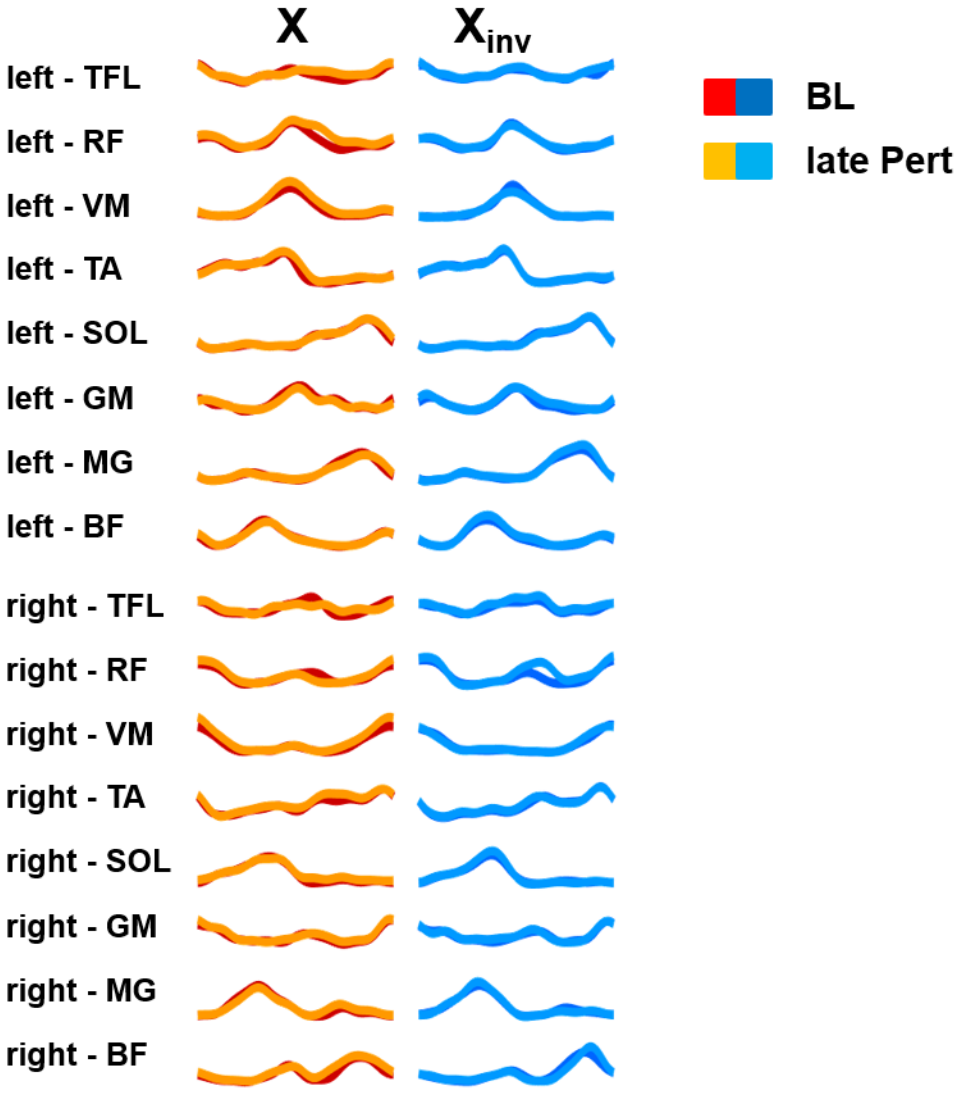

***Figure S5*. EMG Envelopes.** The figure shows the average EMG envelopes during the *BL* (red, blue) and the *late-Pert* (orange, azure) phases for both experiments. To extract the envelopes, the EMG data was first band-pass filtered (50 to 450 Hz) using a 7th-order elliptic filter. The filtered signals were then full-wave rectified and low-pass filtered (cut-off of 5 Hz) using a 7th-order elliptic filter. The changes observed in the single EMG envelopes between the *BL* and the *Pert* phases are, for most channels, minimal.

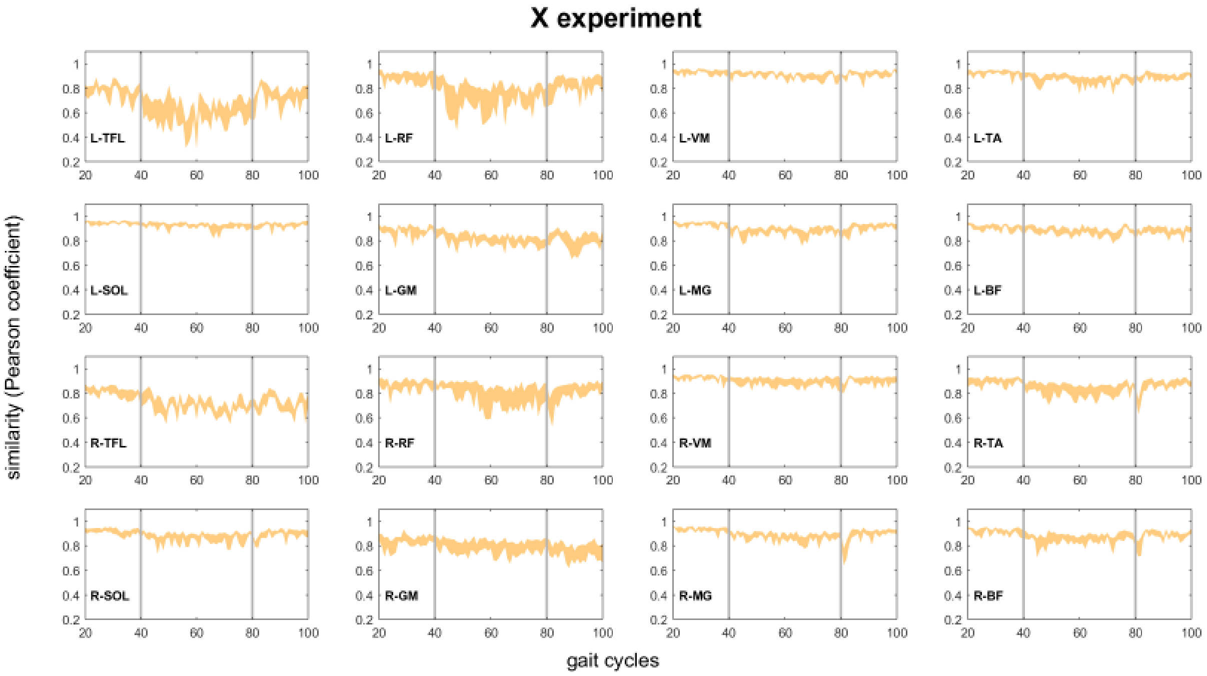

***Figure S6*. Similarity plots derived from the EMG data of the X experiment.** We calculated the similarity between each 2-step segment of the EMG envelopes and the average *BL* envelope for all muscles during the X experiment. Similarity was evaluated using the Pearson correlation coefficient. Each plot shows the average and standard error of the similarity calculated across subjects. Although adaptive behaviors are visible in some of the muscles, adaptation is less clearly identifiable in the EMG data compared to the synergy similarity plots presented in **Figure 5** of the manuscript.

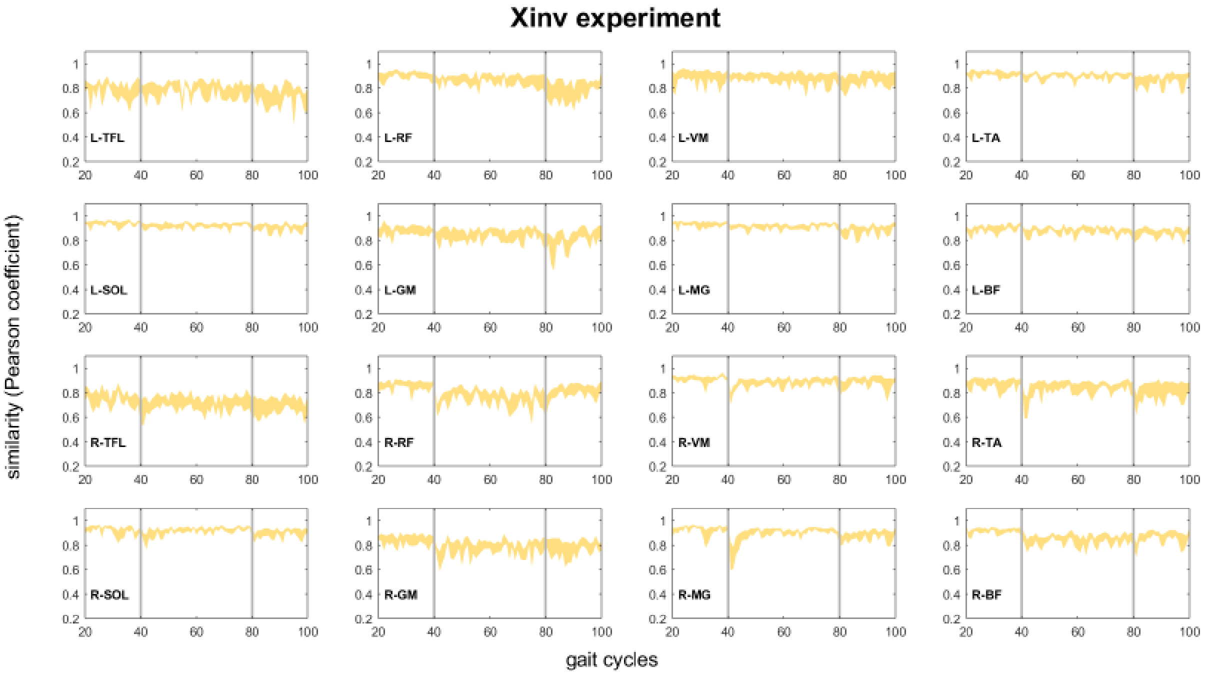

Figure S7. **Similarity plots derived from the EMG data of the X_{inv} experiment.** The figure shows similar plots as per Figure S6 but for the X_{inv} experiment.

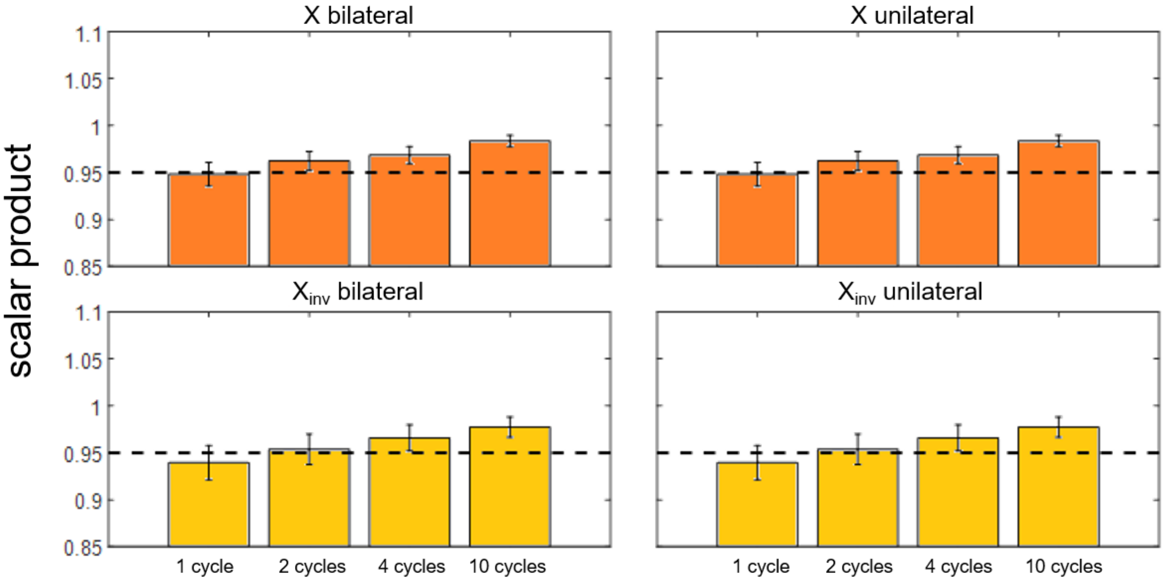

***Figure S8*. Validation of the epochal extraction of muscle synergies for different epoch lengths**. We calculated the normalized dot product between the synergy modules as extracted from all the *BL* data and the *BL* data divided in segments of 1, 2, 4 and 10 gait cycles. The bar plots present the average and standard deviation (across segments and subjects) of the similarity for each segment length for the X (top plots) and X_{inv} (bottom plots) experiments for both the bilateral (left plots) and the unilateral (right plots) analyses. An empirical threshold of 95% similarity is plotted as a dashed line. We demonstrate that synergies extracted using 2-step segments yield a similarity >95% with the ones extracted from the whole *BL* dataset.

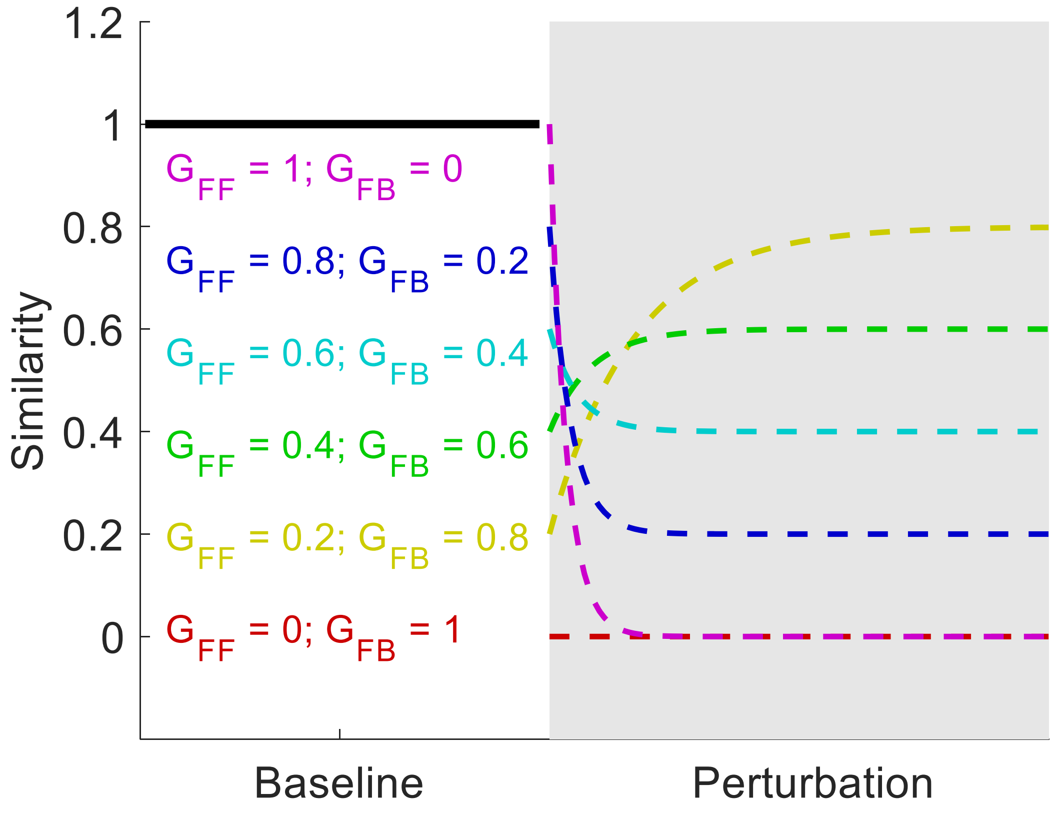

$$G_{FF} * e^{-\beta_{FF}*step} - \gamma_{FF} + 1 - G_{FB} * e^{-\beta_{FF}*step}$$

where

$$\beta_{FF} = \beta * G_{FF}$$

***Figure S9*. Computational model schematically presented in Figure 6A to fit the similarity data**. The equation is meant to model the synergy activation similarity. The model accounts for the contributions of feedforward and feedback components of adaptation to the synergy activation similarity. The feedforward component is represented by an exponential starting from 1 (full similarity, indicating the baseline synergy activation) and decaying towards a final adapted state γ_{FF}. The time constant β_{FF} depends on the overall gain of the feedback response G_{FF} (as expected from previous work[57]). The feedback response is modeled as a step-like response depending on the feedback gain G_{FB}. However, the feedback gain is also multiplied by an exponential with constant β_{FF} (thus establishing a dependence on the feedforward gain G_{FF}) to reflect the fact that the initial feedback response is also affected by the feedforward adaptation, and that final adapted states need to consider also modifications in the motor plan needed to overcome the effects of the feedback response. As a result, when the feedforward gain is 0, the feedback response is exactly step-like. In the plot, $\beta = 0.8$ and $\gamma_{FF} = G_{FF}$. The results of our study suggest that the relative contribution of the two gains is context dependent. Moreover, previous literature[14,57] suggests that both gains also depend on the magnitude of the perturbation.

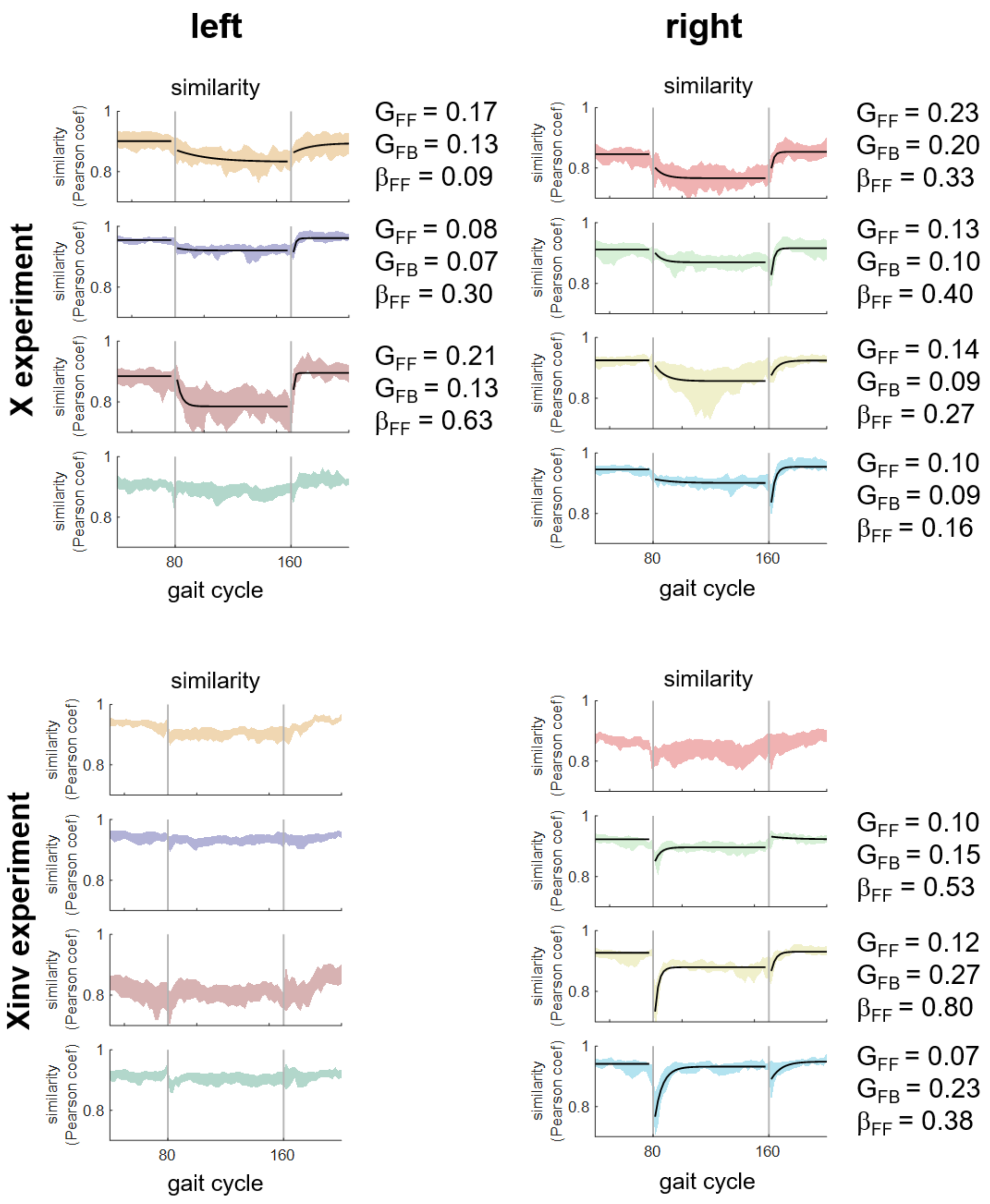

***Figure S10*. Fitting of computational model to similarity data.** The plots show the parameters of the model defined in Figure S9 when fitted to the data presented in Figure 5. For simplicity $\gamma_{FF} = G_{FF}$ in the equation. The results show that adaptation for the X perturbation is mainly characterized by a feedforward response, while that for X_{inv} is mainly characterized by a feedback response.

		Time constant (gait cycles)
X experiment	Pert - S1L	64.4 (34.4, 511.2)
	AE -S1L	37.4 (18.2, 775.3)
	Pert - S2L	20.6 (5.6, 20.6)
	AE -S2L	4.2 (2.3, 23.1)
	Pert - S3L	9.6 (5.6, 34.0)
	AE -S3L	2.3 (0.6, 80.1)
	Pert - S4L	
	AE -S4L	
X_{inv} experiment	Pert - S1L	
	AE -S1L	
	Pert - S2L	
	AE -S2L	
	Pert - S3L	
	AE -S3L	
	Pert - S4L	
	AE -S4L	

		Time constant (gait cycles)
X experiment	Pert - S3R	18.3 (9.4, 292.0)
	AE -S3R	5.4 (2.5, 37.1)
	Pert - S4R	14.8 (8.6, 54.5)
	AE -S4R	5.7 (3.7, 12.6)
	Pert - S1R	21.5 (8.7, 46.6)
	AE -S1R	13.8 (9.2, 27.7)
	Pert - S2R	36.6 (15.6, 107.8)
	AE -S2R	7.7 (5.7, 11.7)
X_{inv} experiment	Pert - S3R	
	AE -S3R	
	Pert - S4R	11.4 (7.2, 27.7)
	AE -S4R	45.9 (14.6, 40.1)
	Pert - S1R	7.5 (5.7, 11.1)
	AE -S1R	9.0 (6.2, 16.6)
	Pert - S2R	15.8 (13.9, 18.4)
	AE -S2R	21.2 (13.8, 46.0)

***Table S1*. Time constants of adaptation derived from the similarity plots shown in Figure 5.** The time constants of adaptation are estimated as 3 times the time constant β (see Equation 3, *Materials and Methods*) of the exponentials fitting the data (i.e. similarity plots). When the data was not modeled in a satisfactory manner by an exponential function, the table does not show a time constant value. Data is shown as the estimated value plus the 95% confidence intervals in parenthesis. It is worth pointing out that the right step-length adaptation observed for the X experiment was marked by a time constant of 13.8 (10.6, 19.9) gait cycles for the *Pert* phase and of 6.0 (4.2, 10.3) gait cycles for the *AE* phase. The step-length adaptation observed for the X_{inv} experiment was marked by a time constant of 9.3 (7.2, 13.3) gait cycles for the *Pert* phase and of 7.3 (4.9, 14.3) gait cycles for the *AE* phase.
